# Perception of brain-computer interface implantation surgery for motor, sensory, and autonomic restoration in spinal cord injury and stroke

**DOI:** 10.3389/fnins.2026.1678175

**Published:** 2026-03-18

**Authors:** Derrick Lin, Tracie Tran, Shravan Thaploo, Jose Gabrielle E. Matias, Joy E. Pixley, Zoran Nenadic, An H. Do

**Affiliations:** 1 Department of Neurology, University of California, Irvine, Irvine, CA, United States; 2 David Geffen School of Medicine at UCLA, Los Angeles, CA, United States; 3 Department of Biomedical Engineering, University of California, Irvine, Irvine, CA, United States; 4 Independent Researcher (formerly at University of California, Irvine), Irvine, CA, United States; 5 Department of Electrical Engineering and Computer Science, University of California, Irvine, Irvine, CA, United States

**Keywords:** brain-computer interface (BCI), electrocorticography (ECoG), functional restoration, neuroethics, survey, spinal cord injury (SCI), stroke rehabilitation, surgical willingness

## Abstract

**Introduction:**

Stroke and spinal cord injury (SCI) can profoundly diminish quality of life across physical and psychosocial domains, with motor and sensory deficits often persisting despite current therapies. Invasive brain-computer interface (BCI) systems, particularly electrocorticography (ECoG)-based approaches, offer a potential means to bypass neural injury and restore function. To inform development and deployment, it is critical to understand candidate users' willingness to adopt such technology and how that willingness relates to their functional goals and rehabilitation priorities.

**Methods:**

We conducted a survey assessing receptiveness to surgical implantation of ECoG grids for BCI use and eliciting participants' rehabilitative goals and perceived priorities across motor and sensory domains. We examined associations between willingness to undergo implantation and (1) the level of functional recovery hypothetically offered, (2) stated rehabilitative priorities, and (3) self-reported disability.

**Results:**

We surveyed 71 participants: stroke (*n* = 33), SCI (*n* = 37), and both stroke and SCI (*n* = 1). Across this cohort, respondents reported a high willingness to undergo surgery for ECoG-based BCI if it could restore basic functions, including upper-extremity control, gait, bowel/bladder function, and sensation. Willingness to pursue implantation showed no correlation with the degree of functional recovery promised by the hypothetical BCI. Likewise, willingness did not correlate with participants' rehabilitative priorities or their level of disability.

**Discussion:**

These findings indicate a strong interest in invasive BCIs even when only basic functions may be restored, independent of disability severity or stated priorities. This suggests that first-generation commercial invasive BCIs with limited functionality may still find receptive users. However, stated interest may not translate to informed surgical consent in real-world contexts, thereby highlighting the risk of overly optimistic expectations. Hence, robust, transparent consent frameworks and balanced communication are essential as invasive BCIs move toward clinical deployment.

## 1 Introduction

Stroke and spinal cord injury (SCI) are debilitating neurological conditions with no current means to reverse functional loss fully. Beyond motor impairment, patients often experience complications such as neurogenic bladder and bowel, urinary tract infections, spasticity, cardiovascular issues, and depression. Given the significant public health impact, interventions that enhance independence and address psychological effects are urgently needed. Brain-computer interface (BCI) technology has recently gained attention as a promising approach to restoring motor, sensory, and possibly autonomic functions in these populations.

BCIs are systems that translate brain signals into commands for external effectors (Shih et al., 2012). In essence, BCIs can potentially enable participants affected by neurological injuries to gain “brain control” of external assistive systems such as a prosthetic limb. Currently, there are several methods for signal acquisition, including scalp electroencephalography (EEG), microelectrode arrays (MEAs), electrocorticography (ECoG), and emerging endovascular approaches (Oxley et al., 2021). MEA-based BCIs have demonstrated remarkable success in motor and sensory restoration demonstrating restoration of reaching, grasping, and somatosensory feedback in individuals with tetraplegia (Ajiboye et al., 2017; Collinger et al., 2013; Bouton et al., 2016; Flesher et al., 2016). Commercial efforts such as Neuralink (Musk and Neuralink, 2019) and Synchron (Oxley et al., 2021) are also seek to advance the field toward clinical viability. It is notable that even current commercial implantable BCI devices utilize epidural or subdural electrode configurations similar to ECoG, further supporting the clinical relevance of our focus on this modality.

In this study, we focused on ECoG-based BCI due to several practical considerations for long-term clinical deployment. ECoG offers a favorable balance between signal quality and invasiveness—providing higher spatial resolution than EEG while avoiding the cortical penetration required by MEAs. Studies have shown that fully implantable ECoG grids exhibit long-term biocompatibility without cortical damage and with sustained neural activity recording (Degenhart et al., 2016). ECoG-based systems have shown growing utilization in implantable BCIs with potentially high clinical impact, including language/speech BCIs (Komeiji et al., 2024; Liu et al., 2023; Metzger et al., 2023), restoration of gait after spinal cord injury (SCI) (Lorach et al., 2023; Lim et al., 2024), and sensory restoration (Lim et al., 2024, 2026). The development of fully implantable and wireless BCI systems may maximize recipient functional independence while long-term biocompatibility would reduce the necessity of repeat procedures or device failure, making ECoG a promising approach for BCI signal acquisition.

Technological potential alone does not ensure adoption. As invasive BCIs approach clinical use, understanding recipients perspectives is essential. Success depends not only on technical efficacy but also on users willingness to undergo surgery and their perceptions of potential benefits. Prior surveys suggest that most participants were aware of BCIs and held positive views, even toward invasive systems (Sattler and Pietralla, 2022; Monasterio Astobiza et al., 2021; Schmid et al., 2021). While positive attitudes support adoption (König et al., 2022), user satisfaction often hinges on whether personal needs are met (Martin et al., 2011). Although ECoG-based BCIs are technically feasible, further research is needed to assess how well they align with users goals for functional restoration. Ultimately, success depends on understanding end-user perceptions—not just developer assumptions.

Previous studies have identified key rehabilitation priorities for individuals with stroke and spinal cord injury (SCI), including motor, autonomic, and cognitive functions (Anderson, 2004; Brown-Triolo et al., 2002; Simpson et al., 2012; Pollock et al., 2012). While these findings highlight broad functional goals, several aspects of stakeholder perception remain underexplored. Specifically, no studies to date have examined how the severity of disability or the perceived potential for functional restoration affects a user's willingness to undergo invasive BCI surgery. Additionally, while motor outcomes are often emphasized, the potential of BCI systems to restore sensory function—and how this is perceived by users—has received limited attention.

In this study, we aimed to elucidate the receptiveness of stroke and SCI participants to invasive ECoG-based BCI technologies and assess their willingness to undergo a prospective surgery for device implantation based on potential motor and sensory restoration. Furthermore, we examined how disability severity, perceived potential functional gains, and rehabilitation priorities influence this willingness. Moreover, this survey also assessed participant attitudes toward both sensory and motor restoration. Finally, we also sought to understand concerns that stroke and SCI participants may have regarding the invasive ECoG-based BCI technology.

## 2 Methods

### 2.1 Participant recruitment

Participants were recruited at the University of California, Irvine (UCI) neurology clinics, UCI occupational and physical therapy clinics, community rehabilitation centers, SCI or stroke outreach events, or via email and support group forums. Inclusion criteria included age >18 and the presence of chronic (>6 months post onset) SCI, stroke, or both. Exclusion criteria included having an injury for less than 6 months or not having the capability to answer the survey questions. Participants were provided with a $10 Amazon gift card at the conclusion of the survey as an incentive. Participants completed the survey electronically through a computer.

### 2.2 Survey development and structure

The survey was designed to determine (1) the importance of functional restoration to SCI and stroke participants at various levels of injury; (2) whether SCI and stroke participants at various levels of injury would consider implantation of an ECoG-based BCI device if varying degrees of motor and sensory functions can be regained; and (3) whether prior knowledge of invasive BCI systems would affect the willingness to undergo surgery for a given restoration.

The survey content was developed by the study team based on clinical experience with stroke and SCI patients. Informational diagrams explaining treatment options were drawn from educational materials similar to those used with patients.

The survey was implemented as an internet-based webform with a combination of multiple-choice and free-response questions, and was designed to take approximately 15 min to complete. Participants either completed the survey remotely or on-site using a provided computer, with an administrator present. To avoid introducing bias in participant responses, survey administrators did not answer any questions outside of technical issues and basic explanations of the questions and/or response options while the participant completed the survey. Participants were allowed to ask questions about BCI technology after completing the survey. Remote participants were given a weblink to respond to the survey. There were five parts to the survey: **Part 1** (question 1–6) inquired about participants' neural injury, including the type and severity of the injury; **Part 2** (question 7–8) established the participant's pre-existing familiarity with BCI technology; **Part 3** (question 9–23) determined the participant's willingness to undergo implantation of a BCI device given that certain neurological function could be restored; **Part 4** (question 24–29) inquired about additional functions that participants would like restored beyond those previously mentioned; it also assessed the likelihood of undergoing surgery to implant a BCI system for these functions and gathered any concerns regarding the implantation process; **Part 5** (question 30–35) collected demographic information, e.g., age, gender, and other social factors. Each part is described in further detail below. A copy of the survey can be found in the Supplementary material. This study was reviewed by the University of California, Irvine Institutional Review Board (IRB) and determined to be exempt. Written informed consent was obtained from all participants prior to participation, in accordance with institutional and federal guidelines.

To minimize unusable data, participants were given the explicit option to select “Don't know/Decline to answer” for most questions. Where appropriate, skip logic was used to route participants past questions irrelevant to their injury type (e.g., participants indicating paraplegia were not asked about arm function). If a participant skipped a question without selection, the response was coded as “missing.” Questions skipped due to programmed logic were coded as “not applicable” (NA). These categories (DK/REF, missing, NA) were tracked separately in the analysis.

**Part 1** of the survey was designed to establish participants' type of neural injury (SCI, stroke, or both), the extent of the injury (if known, ASIA Scale for SCI, Modified Rankin Scale for stroke), and the type of prostheses participants were currently using. This was accomplished with multiple choice questions for each of the above with the inclusion of “Don't know/Decline to Answer” option to prevent inaccurate guesses if participants were uncertain. Responses marked as “Don't know/Decline to answer” were retained but excluded from analyses that specifically required those data points (e.g., ASIA or Rankin stratification).

**Part 2** of the survey asked participants whether they had prior familiarity with ECoG BCI technology to determine whether prior knowledge of BCI impacted the participants' responses regarding their willingness to undergo surgery for a certain functional restoration. Regardless of the response, participants were given written and visual descriptions of a hypothetical ECoG-based BCI implant (see Supplementary material for complete details) and the mechanism by which it can potentially restore function. It then detailed the necessity for the surgical implantation of an ECoG electrode grid to record brain wave activities so that all participants understand the procedure involved.

**Part 3** of the survey was intended to establish participants' perception on the importance of regaining upper extremity motor, gait, and sensation function, as well as whether the participants were willing to undergo BCI implantation procedures if varying degrees of each of the above functions can be restored. For upper extremity function, the increments of function restored included “basic grasp and release,” “fine control of fingers along with basic grasp and release” and “fine control of arms” along with the previous two functions. For walking, increments included “Ability to stand,” “Ability to walk at constant speed,” “Ability to walk at various speeds,” and “Ability to make turns along with walking at various speeds.” Finally, participants were asked about their willingness to undergo surgery to restore a particular sensation, specifically regarding legs, arms, hands and fingers, and bladder fullness.

These questions all used the same standard unipolar response options for extent (i.e., very, moderately, slightly, not at all) to minimize cognitive load for participants and to allow for comparable analyses across questions. These items were not combined into scales, as we are not positing underlying constructs of “importance” or “willingness” that apply to disparate types of conditions or treatments.

**Part 4** of the survey asked if there were any other neurological functions not mentioned in the survey that the participant would like restored and the likelihood they would undergo surgery to implant a BCI system for such functions. Part 4 also inquired about any concerns participants had with a prospective implantation of the BCI system.

**Part 5** of the survey was composed of demographic questions (i.e., age, gender, level of education, current occupation, current living situation, and household income).

### 2.3 Analytic methods

#### 2.3.1 Participant classification

Responses to Part 1 (questions 1–6) of the survey—covering injury type (SCI, stroke, or both), nature of impairment (e.g., tetraplegia/paraplegia), ASIA Impairment Scale, level of injury, Modified Rankin Scale, and current mobility aid—served as the basis for classifying participants into appropriate subgroups for the analyses presented in subsequent sections.

Participants who selected “Don't know/Decline to answer” were excluded only from analyses where their response was required for subgrouping (e.g., ASIA or Rankin classification). Participants who were programmatically skipped past a question were coded as “not applicable” and retained in other analyses. Completely missing responses were rare and handled as missing data.

#### 2.3.2 Effect of injury severity on perceived importance of functional restoration

Responses to **Part 3** (questions 9, 13, and 18) of the survey were used to assess the distribution of participant's perceived importance toward regaining motor and sensory functions. Relative preference (Equation 1) was used to quantify how much more (or less) likely participants with a given characteristic (e.g., prior knowledge of BCI or injury severity) were to express a particular response compared to participants without that characteristic. In most cases, this refers to variations in the level of a specific characteristic, such as differing degrees of prior knowledge about BCI or varying severity of injury. This measure captures the ratio of probabilities between groups, analogous to relative risk in epidemiological studies, and allows us to assess whether certain factors influence preferences or willingness. To handle instances where a cell count was zero (which would otherwise result in undefined or extreme scores), the Haldane-Anscombe correction was applied by adding 0.5 to all cell counts in the 2 × 2 contingency table. This adjustment provides more stable estimates of the relative preference, particularly in small sample sizes (Weber et al., 2020). In the case of the participant's perceived importance of functional restoration, the null hypothesis was that the severity of the injury does not change the important/unimportant ratio. A 95% confidence interval was also calculated using Equation 3.

The equation for relative preference (*RP*):


$$\begin{array}{l} RP=\frac{a{\left(a+c\right)}^{-1}}{b{\left(b+d\right)}^{-1}}=\frac{a\left(b+d\right)}{b\left(a+c\right)} \end{array}$$ (1)

The equation for standard error (*SE*) of *RP*:


$$\begin{array}{l} SE\left(\log\left(RP\right)\right)=\sqrt{\frac{c}{a\left(a+c\right)}+\frac{d}{b\left(b+d\right)}} \end{array}$$ (2)

The equation for the 1−α confidence interval (*CI*) of *RP*:


$$\begin{array}{l} CI_{1-\alpha }\left(\log\left(RP\right)\right)=\log\left(RP\right)+SE\left(\log\left(RP\right)\right)\times z_{\alpha } \end{array}$$ (3)

where *a*, *b*, *c*, and *d* are defined in Table 1 and *z*_α_ is defind as the standard score for level of significance α.

**Table 1:** Variable definitions for relative preference calculation (Equations 1, 2).

**Willingness level**	**More severe (Rankin 4, 5; ASIA A, B)**	**Less severe (Rankin 2, 3; ASIA C, D)**
More willing (Very/moderately likely)	a	b
Less willing (Slightly/not at all likely)	c	d

#### 2.3.3 Effect of levels of restoration on willingness to undergo ECoG-BCI implantation

Responses to **Part 3** (questions 10–12, 14–17, and 19–23) of the survey were used to determine whether offering more functional capabilities in the ECoG-based BCI devices would lead to an increase in participants' willingness to undergo surgery to implant the system for upper-extremity motor, lower extremity motor, and sensation functions. Relative preferences to undergo surgery with respect to injury severity to restore the aforementioned functions were calculated using Equation 1. The null hypothesis was that the severity of the injury does not change the willing/unwilling ratio. A 95% confidence interval was also calculated.

#### 2.3.4 Effect of perceived importance of functional restoration on willingness to undergo ECoG-BCI implantation

Responses to **Part 3** (questions 9–23) of the survey were used to determine whether an increase in the perceived importance to regain upper-extremity motor, lower extremity motor, and sensation functions would also increase willingness to undergo surgery for the appropriate restorations. Relative preference to undergo surgery with respect to perceived importance of the aforementioned functions were calculated using Equation 1. The null hypothesis was that the perceived importance of a given function does not change the willing/unwilling ratio. A 95% confidence interval was also calculated using Equation 3.

#### 2.3.5 Effect of prior knowledge on willingness to undergo ECoG-BCI implantation

Responses to **Part 2** (question 8) of the survey combined with **Part 3** (questions 12, 17, 19–21, and 23) was used to determine whether prior ECoG BCI knowledge influenced the relative preference to undergo surgery to restore upper/lower extremity motor or sensation functions. Relative preference (Equation 1) is used to assess whether prior knowledge of implantable BCI influences a participant's willingness to undergo surgery. The null hypothesis is that given prior knowledge of BCI, the participant is equally as willing to undergo surgery for functional restoration compared to someone without BCI knowledge. A 95% confidence interval was also calculated using Equation 3.

#### 2.3.6 Effect of demographics on BCI receptiveness

*Post-hoc* analyses examined whether demographic variables from **Part 5** of the survey (age, gender, education level, and household income) influenced perceived importance of functional restoration and willingness to undergo surgery. Demographic variables were dichotomized as follows: age (younger < 55 years vs. older ≥55 years), gender (male vs. female), education (higher ≥college degree vs. lower ≤ high school), and household income (higher ≥$100,000 vs. lower < $40,000). Participants with intermediate values or missing responses were excluded from the relevant comparison. Relative preference was calculated using Equation 1, with 95% confidence intervals calculated using Equation 3. The null hypothesis was that demographic characteristics do not affect the relative preference.

#### 2.3.7 Additional functional restoration

Responses to **Part 4** (questions 24–26) of the survey were analyzed to assess participants' interest in restoring additional functions beyond those listed in earlier sections, as well as their willingness to undergo surgery for such restorations. Question 25 included an open-ended text box in which participants could freely describe any additional functions they wished to regain. Responses were reviewed and categorized into thematic groups.

#### 2.3.8 Concerns

Responses to **Part 4** (question 28) of the survey was analyzed to identify participants' concerns about undergoing a prospective ECoG-based BCI implantation. The number of participants expressing a specific concern was normalized and presented as a percentage of the total survey participants.

## 3 Results

### 3.1 Participant classification

A total of 71 participants responded to the survey. The participant injury profile and demographic information are summarized in Table 2. Of the 71 participants, 4 did not provide sufficient information on the ASIA Impairment Scale and were excluded from subgroup analyses involving injury severity. This resulted in a final analytic sample of 12 with tetraplegia and 15 with paraplegia.

**Table 2:** Characteristics of survey participants.

**Category**	**Group**	**Total (%)**	**SCI (%)**	**Stroke (%)**	**Both (%)**
Type of injury	All participants	71	33	37	1
	SCI	34 (47.89)	33 (100.00)	-	1 (100.00)
	Stroke	38 (53.52)	-	37 (100.00)	1 (100.00)
Type of SCI deficit	Tetraplegia	12 (16.90)	11 (33.33)	-	1 (100.00)
	Paraplegia	15 (21.13)	15 (45.45)	-	0 (0.00)
	Don't know/decline to answer	1 (1.41)	1 (3.03)	-	0 (0.00)
	Not applicable	6 (8.45)	6 (18.18)	-	0 (0.00)
Level of SCI impairment	Cervical	17 (23.94)	16 (48.48)	-	1 (100.00)
	Thoracic	3 (4.23)	3 (9.09)	-	0 (0.00)
	Lumbar	3 (4.23)	3 (9.09)	-	0 (0.00)
	Sacral	0 (0.00)	0 (0.00)	-	0 (0.00)
	Don't know/decline to answer	5 (7.04)	5 (15.15)	-	0 (0.00)
	Not applicable	6 (8.45)	6 (18.18)	-	0 (0.00)
ASIA scale	Grade A	7 (9.87)	7 (21.21)	-	0 (0.00)
	Grade B	12 (16.90)	12 (36.36)	-	0 (0.00)
	Grade C	7 (9.87)	6 (18.18)	-	1 (100.00)
	Grade D	4 (5.63)	4 (12.12)	-	0 (0.00)
	Grade E	0 (0.00)	0 (0.00)	-	0 (0.00)
	Don't know/decline to answer	4 (5.63)	4 (12.12)	-	0 (0.00)
Modified rankin scale	0	0 (0.00)	-	0 (0.00)	0 (0.00)
	1	0 (0.00)	-	0 (0.00)	0 (0.00)
	2	3 (4.23)	-	3 (8.11)	0 (0.00)
	3	11 (15.49)	-	11 (29.73)	0 (0.00)
	4	11 (15.49)	-	11 (29.73)	0 (0.00)
	5	12 (16.90)	-	11 (29.73)	1 (100.00)
	Don't know/decline to answer	1 (1.41)	-	1 (2.70)	0 (0.00)
Mobility assistance	Walking aid	11 (15.49)	3 (9.09)	8 (21.62)	0 (0.00)
	Wheelchair	15 (21.13)	11 (33.33)	3 (8.11)	1 (100.00)
	Other	1 (1.41)	1 (3.03)	0 (0.00)	0 (0.00)
	Don't know/decline to answer	0 (0.00)	0 (0.00)	0 (0.00)	0 (0.00)
	Not applicable	44 (61.97)	18 (54.55)	26 (70.27)	0 (0.00)
Age	18–24	9 (12.68)	9 (27.27)	0 (0.00)	0 (0.00)
	25–34	11 (15.49)	9 (27.27)	1 (2.70)	1 (100.00)
	35–44	5 (7.04)	3 (9.09)	2 (5.41)	0 (0.00)
	45–54	7 (9.86)	2 (6.06)	5 (13.51)	0 (0.00)
	55–64	18 (25.35)	4 (12.12)	14 (37.84)	0 (0.00)
	65–74	14 (19.72)	3 (9.09)	11 (29.73)	0 (0.00)
	75 or older	4 (5.63)	1 (3.03)	3 (8.11)	0 (0.00)
	Don't know/Decline to answer	1 (1.41)	1 (3.03)	0 (0.00)	0 (0.00)
	Not applicable	2 (2.82)	1 (3.03)	1 (2.70)	0 (0.00)
Gender	Male	48 (67.61)	22 (66.67)	25 (67.57)	1 (100.00)
	Female	18 (25.35)	8 (24.24)	10 (27.03)	0 (0.00)
	Other	1 (1.41)	1 (3.03)	0 (0.00)	0 (0.00)
	Decline to answer	1 (1.41)	0 (0.00)	1 (2.70)	0 (0.00)
	Not applicable	3 (4.23)	2 (6.06)	1 (2.70)	0 (0.00)
Level of education	Some high school or less	4 (5.63)	3 (9.09)	1 (2.70)	0 (0.00)
	High school diploma or equivalent	11 (15.49)	5 (15.15)	6 (16.22)	0 (0.00)
	Some college or a two year degree	21 (29.58)	10 (30.30)	11 (29.73)	0 (0.00)
	College graduate	18 (25.35)	8 (24.24)	9 (24.32)	1 (100.00)
	Advanced degree (Master's, JD, MD, etc.)	14 (19.72)	6 (18.18)	8 (21.62)	0 (0.00)
	Don't know/decline to answer	1 (1.41)	0 (0.00)	1 (2.70)	0 (0.00)
	Not applicable	2 (2.82)	1 (3.03)	1 (2.70)	0 (0.00)
Living situation	Nursing facility + Full time assistance	0 (0.00)	0 (0.00)	0 (0.00)	0 (0.00)
	Nursing facility + Partial assistance	2 (2.82)	0 (0.00)	2 (5.41)	0 (0.00)
	Home + supportive living service	6 (8.45)	4 (12.12)	2 (5.41)	0 (0.00)
	Home + somebody else	34 (47.89)	15 (45.45)	18 (48.65)	1 (100.00)
	Home + no assistance	21 (29.58)	7 (21.21)	14 (37.84)	0 (0.00)
	Don't know/Decline to answer	0 (0.00)	0 (0.00)	0 (0.00)	0 (0.00)
	Not applicable	8 (11.27)	7 (21.21)	1 (2.70)	0 (0.00)
Annual income	$9,999 or less	9 (12.68)	4 (12.12)	4 (10.81)	1 (100.00)
	$10,000–$19,999	3 (4.23)	2 (6.06)	1 (2.70)	0 (0.00)
	$20,000–$39,999	11 (15.49)	5 (15.15)	6 (16.22)	0 (0.00)
	$40,000–$59,999	12 (16.90)	4 (12.12)	8 (21.62)	0 (0.00)
	$60,000–$69,999	5 (7.04)	2 (6.06)	3 (8.11)	0 (0.00)
	$70,000–$99,999	2 (2.82)	1 (3.03)	1 (2.70)	0 (0.00)
	$100,000–$199,999	11 (15.49)	6 (18.18)	5 (13.51)	0 (0.00)
	$200,000 or higher	1 (1.41)	1 (3.03)	0 (0.00)	0 (0.00)
	Don't know/decline to answer	15 (21.13)	7 (21.21)	8 (21.62)	0 (0.00)
	Not applicable	2 (2.82)	1 (3.03)	1 (2.70)	0 (0.00)

### 3.2 Effect of injury severity on perceived importance of functional restoration

**Figure 1:**
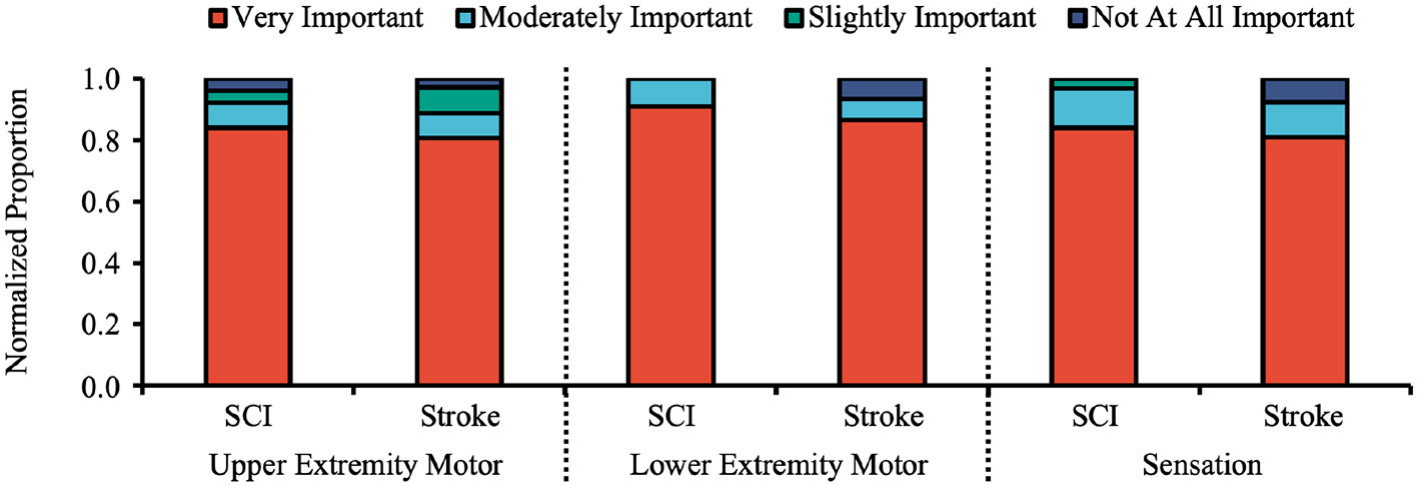
Importance of regaining motor and sensory functions to stroke and SCI participants as a normalized proportion, excluding “Does Not Apply” and “Don't Know/Decline” (included in Supplementary Figure 1). The majority (>80%) of both stroke and SCI participants expressed that regaining upper extremity motor function, lower extremity motor function, and sensation were all “Very Important.”

Overall, SCI participants perceived lower-extremity motor function to be more important than upper-extremity motor function and sensation/bladder functions; Stroke participants perceived both upper- and lower-extremity motor functions to be slightly more important than sensation/bladder functions (Figure 1). Additionally, a larger proportion of SCI participants considered sensory function to be very important compared to stroke participants.

**Figure 2:**
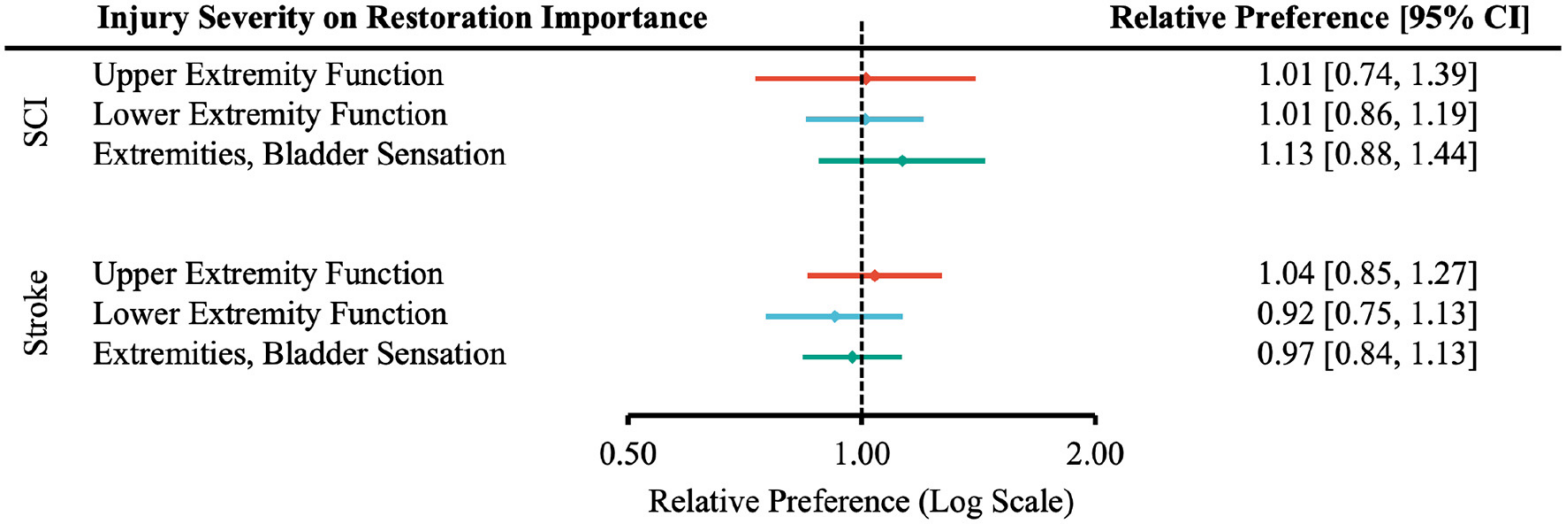
Forest plot of the relative preferences on whether injury severity influenced participants' perceived importance of functional restoration. According to Table 1, the number of participants responding *Very Important* and *Moderately Important* is placed in the “More Willing” group; *Slight Important* and *Not at all Important* in the “Less Willing” group; *ASIA A, B* or *Rankin 4, 5* in the “More Severe” group; *ASIA C, D* or *Rankin 2, 3* in the “Less Severe” group.

The severity of disability did not significantly influence the perceived importance of restoring upper extremity motor, lower extremity motor, or sensory function (no statistically significant differences observed at the 95% confidence level, Figure 2).

**Figure 3:**
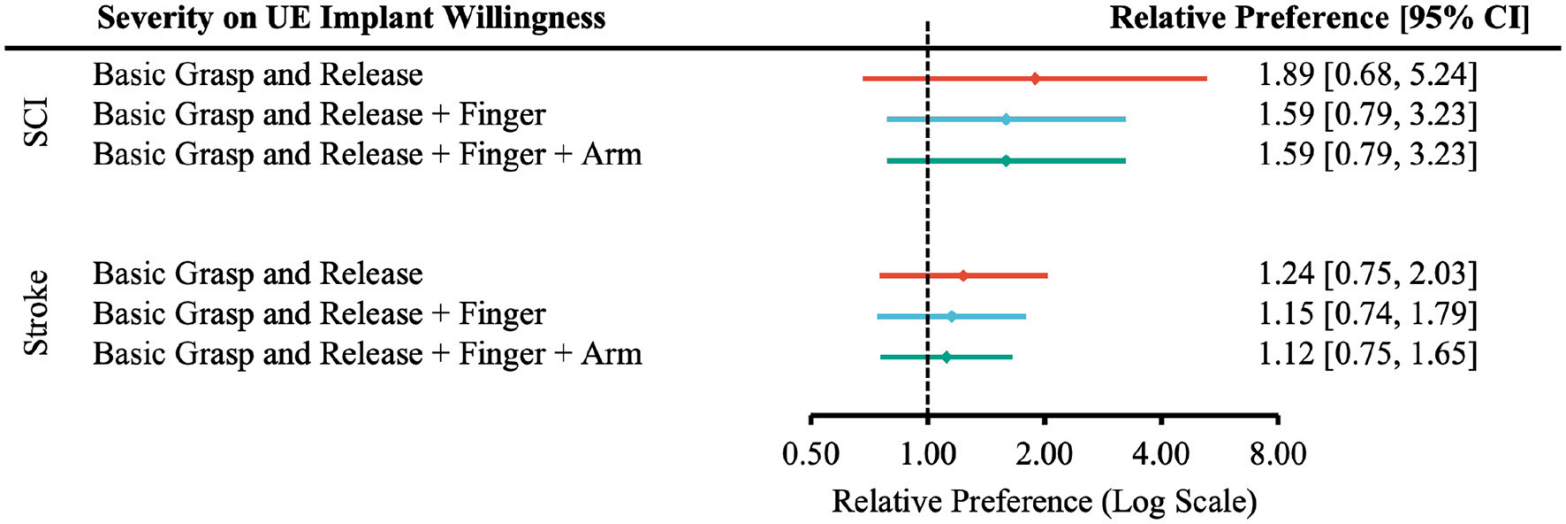
Forest plot of the relative preference to undergo surgery to restore upper-extremity functions with respect to injury severity. According to Table 1, the number of participants indicated *Very Likely* and *Moderately Likely* is placed in the “More Willing” group; *Slight Likely* and *Not at all Likely* in the “Less Willing” group; *ASIA A, B* or *Rankin 4, 5* in the “More Severe” group; *ASIA C, D* or *Rankin 2, 3* in the “Less Severe” group.

**Figure 4:**
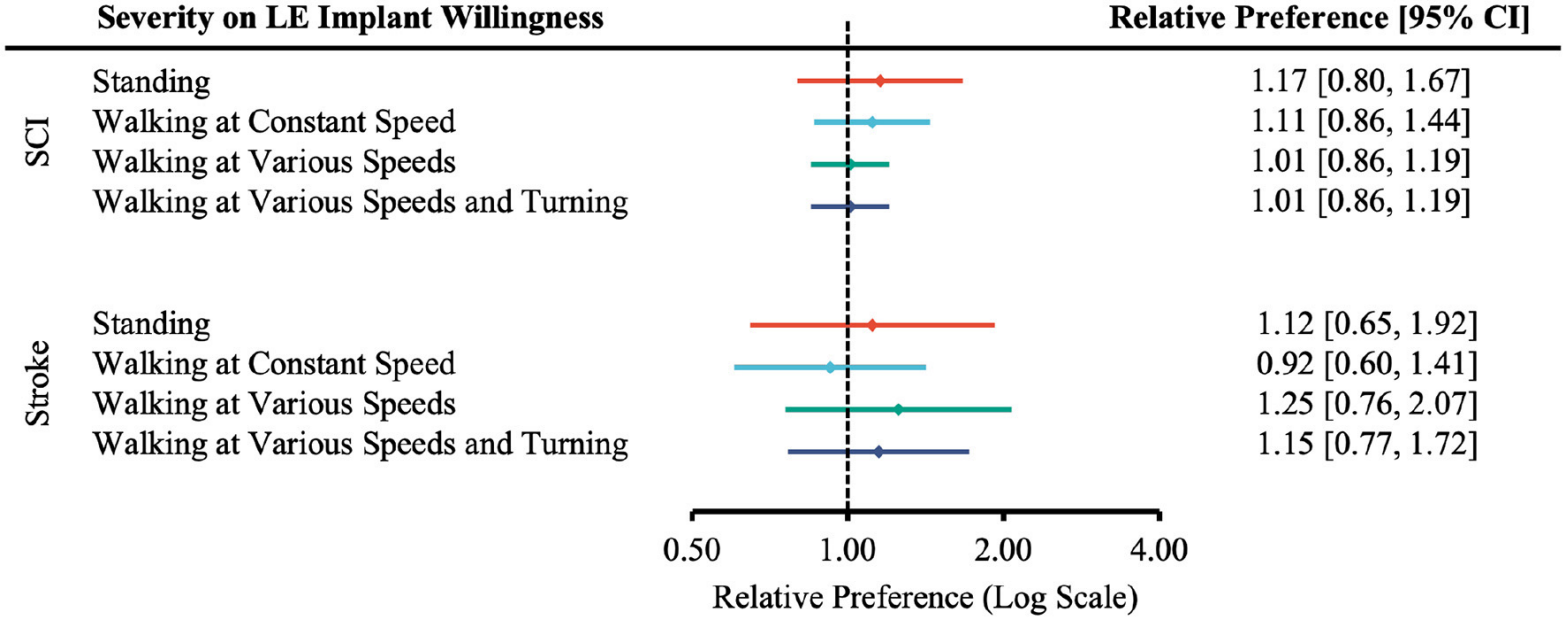
Forest plot of the relative preference to undergo surgery to restore lower-extremity functions with respect to injury severity. According to Table 1, the number of participants indicated *Very Likely* and *Moderately Likely* is placed in the “More Willing” group; *Slight Likely* and *Not at all Likely* in the “Less Willing” group; *ASIA A, B* or *Rankin 4, 5* in the “More Severe” group; *ASIA C, D* or *Rankin 2, 3* in the “Less Severe” group.

**Figure 5:**
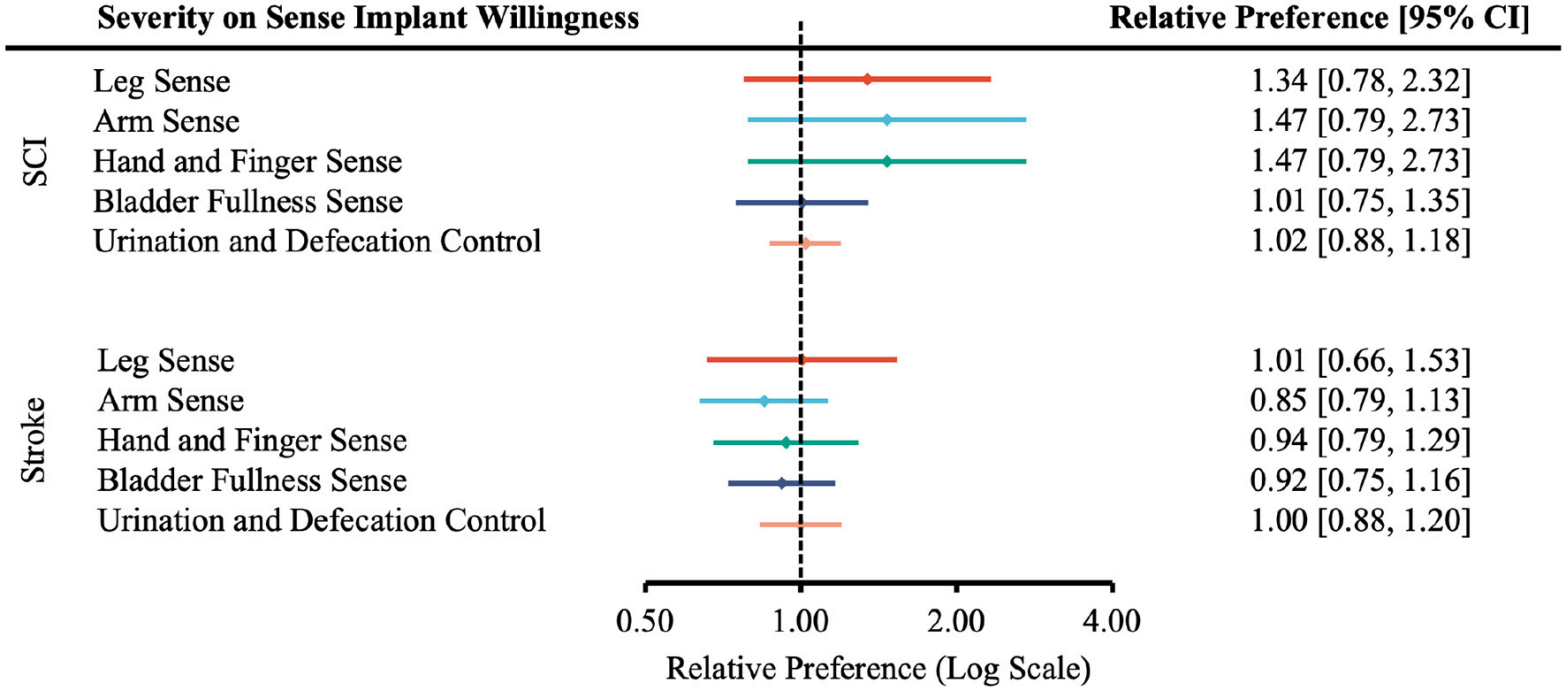
Forest plot of the relative preference to undergo surgery to restore sensory and autonomic functions with respect to injury severity. According to Table 1, the number of participants indicated *Very Likely* and *Moderately Likely* is placed in the “More Willing” group; *Slight Likely* and *Not at all Likely* in the “Less Willing” group; *ASIA A, B* or *Rankin 4, 5* in the “More Severe” group; *ASIA C, D* or *Rankin 2, 3* in the “Less Severe” group.

The relative preference for willingness to undergo surgery based on the degree of injury was not statistically significant (Figures 3–5).

### 3.3 Willingness to undergo ECoG-BCI implantation

**Figure 6:**
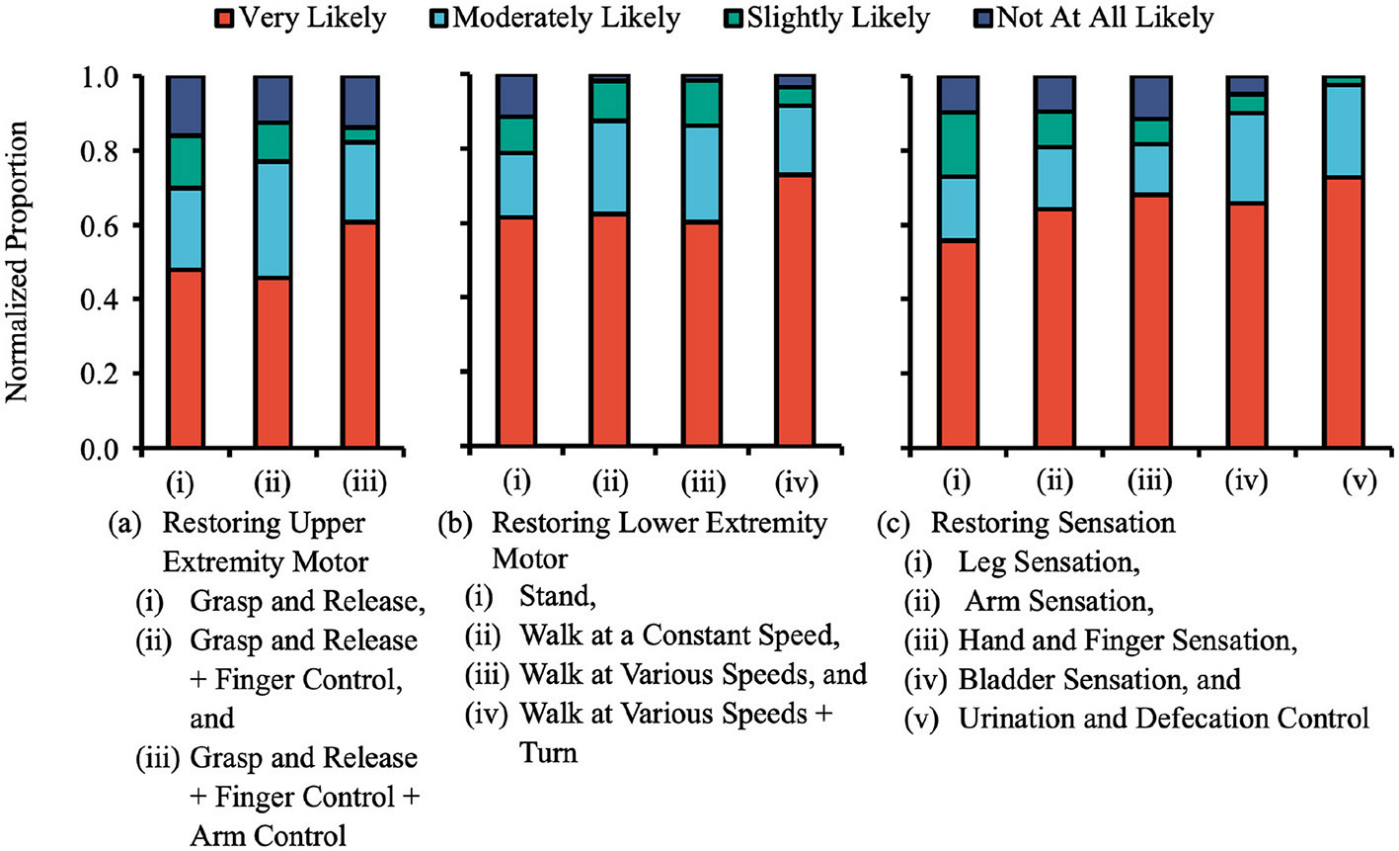
Participant willingness to undergo surgery to implant BCI system at various degrees of function restored. **(a)** Upper extremity motor function. **(b)** Lower extremity motor function. **(c)** Sensation. Participants were asked to indicate their level of disability as well as how important regaining upper extremity motor function, lower extremity motor function, and sensation were to them. Participants' responses were plotted as a sample size-normalized stacked bar graph of those who red that each function was “very important,” “moderately important,” “slightly important,” “not at all important,” or “does not apply.” The majority of participants were at least moderately willing to undergo surgery to implant BCI systems for even basic levels of functional restoration.

The majority of participants responded “Very likely” or “Moderately likely” when asked about their willingness to undergo surgery for an ECoG-based BCI to restore upper extremity motor, lower extremity motor, sensation, and bladder functions (Figure 6). Specifically, for upper extremity function, 70% of participants stated they would be “Very likely” or “Moderately likely” to undergo surgery to restore “Grasp and Release,” 77% for “Grasp and Release + Fine Finger Control,” and 82% for “Grasp and Release + Fine Finger Control + Fine Arm Control.” For lower extremity function, 79% of participants stated they would be “Very likely” or “Moderately likely” to undergo surgery to restore “Stand,” 88% for “Walk at a Constant Speed,” 86% for “Walk at Various Speeds,” and 92% for “Walk at Various Speeds + Turn.” For sensation and urination/defecation control 73% of participants stated they would be “Very likely” or “Moderately likely” to undergo surgery to restore “Leg Sensation,” 81% for “Arm Sensation,” 82% for “Hand and Finger Sensation,” 90% for “Bladder Sensation,” and 98% for “Urination and Defecation Control.”

### 3.4 Effect of levels of restoration on willingness to undergo ECoG-BCI implantation

Responses did not significantly differ based on the degree of functionality restored (Figure 6 illustrates this for upper extremity motor functions).

### 3.5 Effect of perceived importance of functional restoration on willingness to undergo surgery

**Figure 7:**
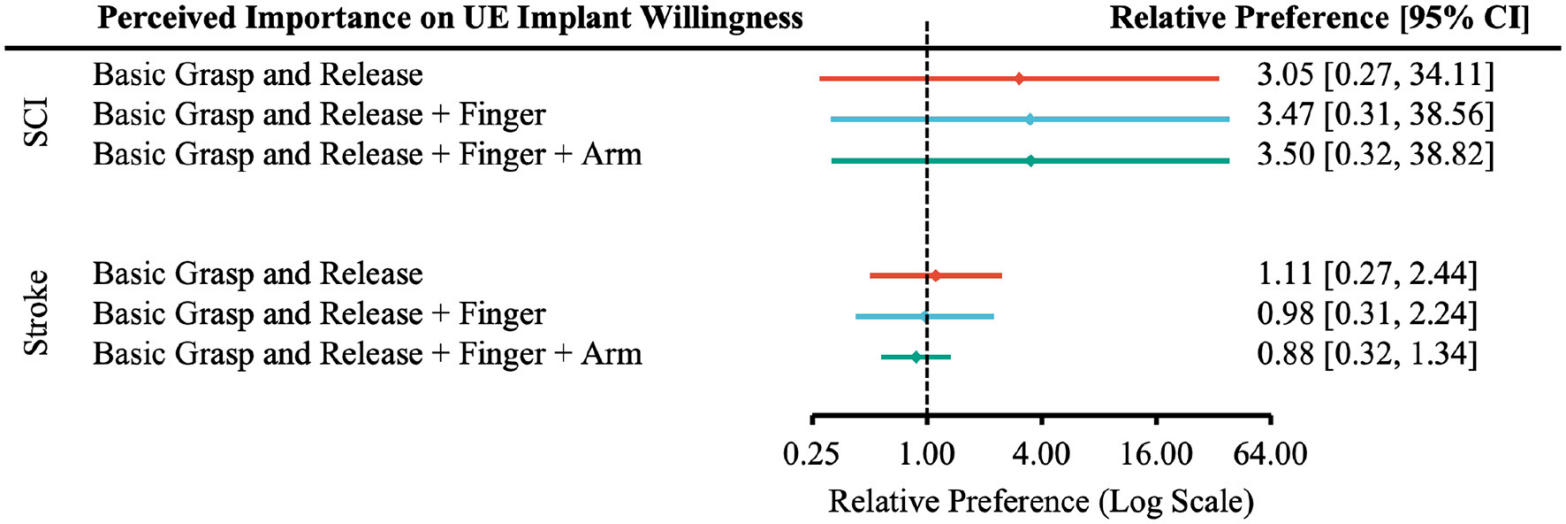
Forest plot of the relative preference to undergo surgery to restore upper-extremity functions with respect to perceived importance of functional restoration. According to Table 1, the number of participants indicated *Very Likely* and *Moderately Likely* is placed in the “More Willing” group; *Slight Likely* and *Not at all Likely* in the “Less Willing” group; *Very Important* and *Moderately Important* in the “More Important” group; *Slight Important* and *Not at all Important* in the “Less Important” group.

**Figure 8:**
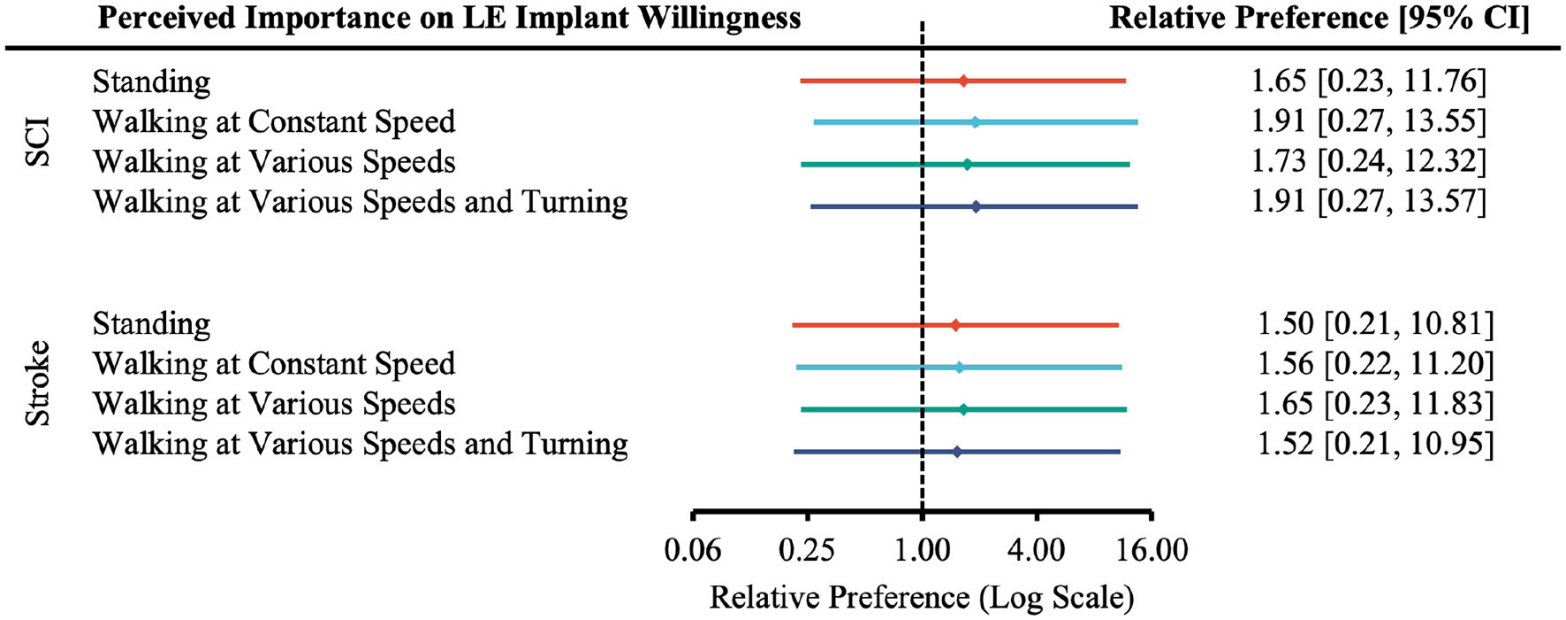
Forest plot of the relative preference to undergo surgery to restore lower-extremity functions with respect to perceived importance of functional restoration. According to Table 1, the number of participants indicated *Very Likely* and *Moderately Likely* is placed in the “More Willing” group; *Slight Likely* and *Not at all Likely* in the “Less Willing” group; *Very Important* and *Moderately Important* in the “More Important” group; *Slight Important* and *Not at all Important* in the “Less Important” group.

**Figure 9:**
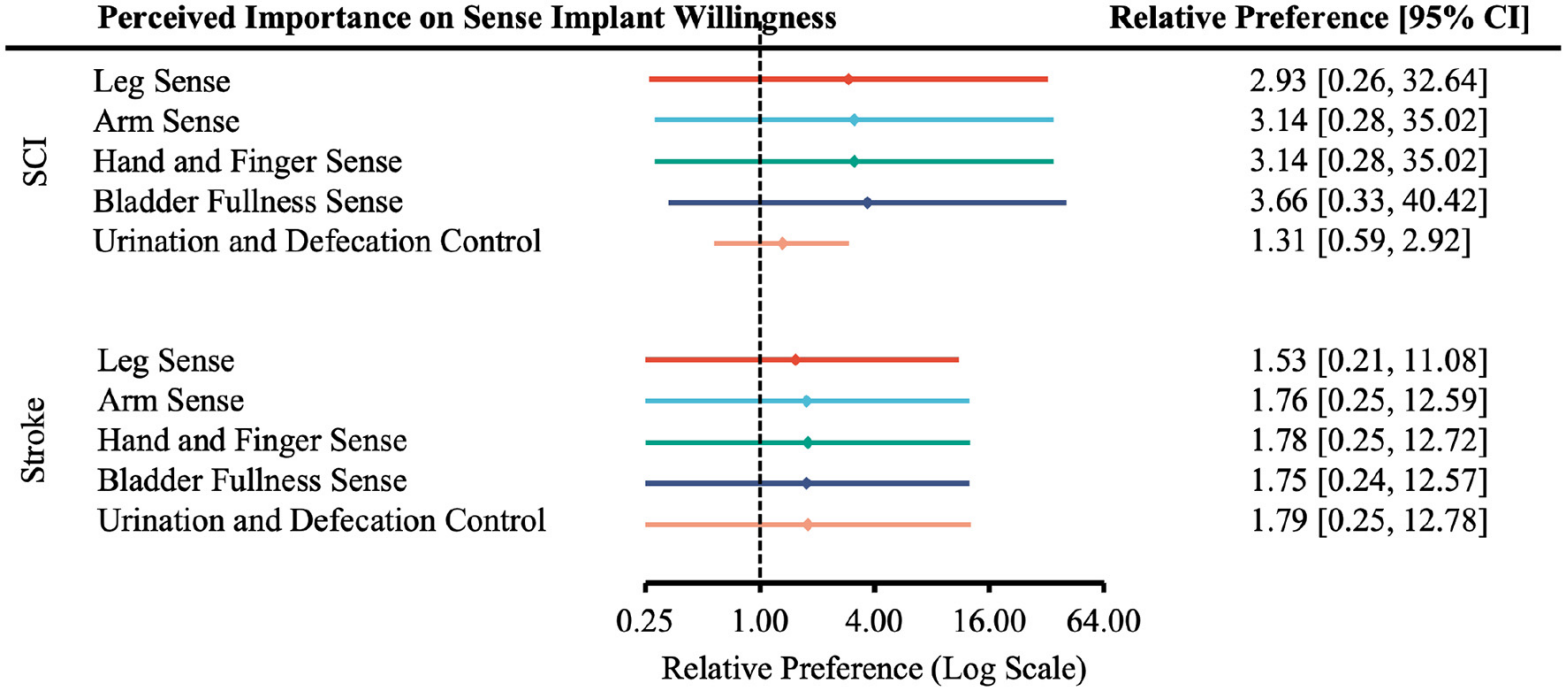
Forest plot of the relative preference to undergo surgery to restore sensory and autonomic functions with respect to perceived importance of functional restoration. According to Table 1, the number of participants indicated *Very Likely* and *Moderately Likely* is placed in the “More Willing” group; *Slight Likely* and *Not at all Likely* in the “Less Willing” group; *Very Important* and *Moderately Important* in the “More Important” group; *Slight Important* and *Not at all Important* in the “Less Important” group.

In all levels of functional restoration, most applicable participants reported “Very Likely” to undergo surgery and rated function restoration as “Very Important” (Supplementary Figures 6, 7). The effect of perceived importance of functional restoration on the relative preference for willingness to undergo surgery was not statistically significant in any case (Figures 7–9).

### 3.6 Effect of prior knowledge on willingness to undergo ECoG-BCI implantation

**Figure 10:**
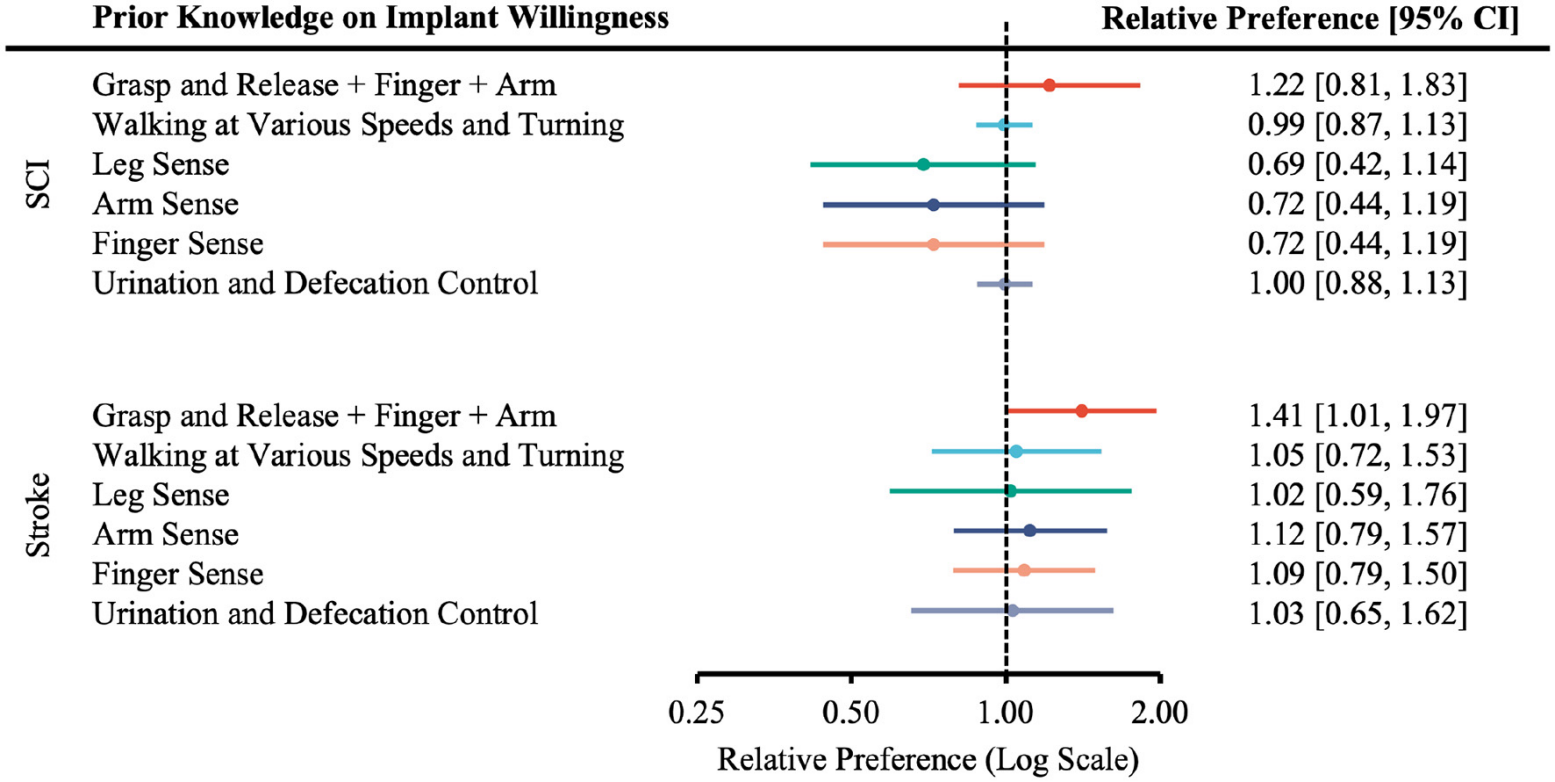
Relative willingness of participants to undergo surgery for device implantation to restore a particular function with or without prior knowledge. The number of participants with prior BCI knowledge is placed in the intervention group; without prior knowledge in the control group; participants that are more willing to undergo surgery in the event group; less willing in the non-event group.

Prior knowledge of BCI technology did not influence willingness to undergo surgery to restore functions with one exception (Figure 10). Specifically, stroke participants with prior knowledge of BCI technology expressed more willingness to undergo surgical implantation of ECoG-based BCIs to restore a combination of arm, grasp/release, and finger control function.

### 3.7 Effect of demographics on BCI receptiveness

Additional *post-hoc* analyses examined whether demographic variables (age, gender, education, household income) influenced perceived importance of functional restoration and willingness to undergo surgery (Supplementary Table 1). Higher household income was significantly associated with greater willingness to undergo surgery for sensory restoration (Table 3). No other demographic associations reached statistical significance.

**Table 3:** Significant demographic associations with willingness to undergo surgery for sensory restoration.

**Outcome**	**RR**	**95% CI**	**Interpretation**
Leg sensation	1.46	[1.08, 1.98]	High income more willing
Arm sensation	1.42	[1.04, 1.93]	High income more willing
Hand/finger sensation	1.36	[1.04, 1.78]	High income more willing

Higher household income was associated with greater willingness across all sensory restoration outcomes.

RR, Relative Risk (High income ≥$100k vs. Low income < $40k).

Significant if 95% CI excludes 1.0. Full results in Supplementary Table S1.

### 3.8 Additional functional restoration

**Figure 11:**
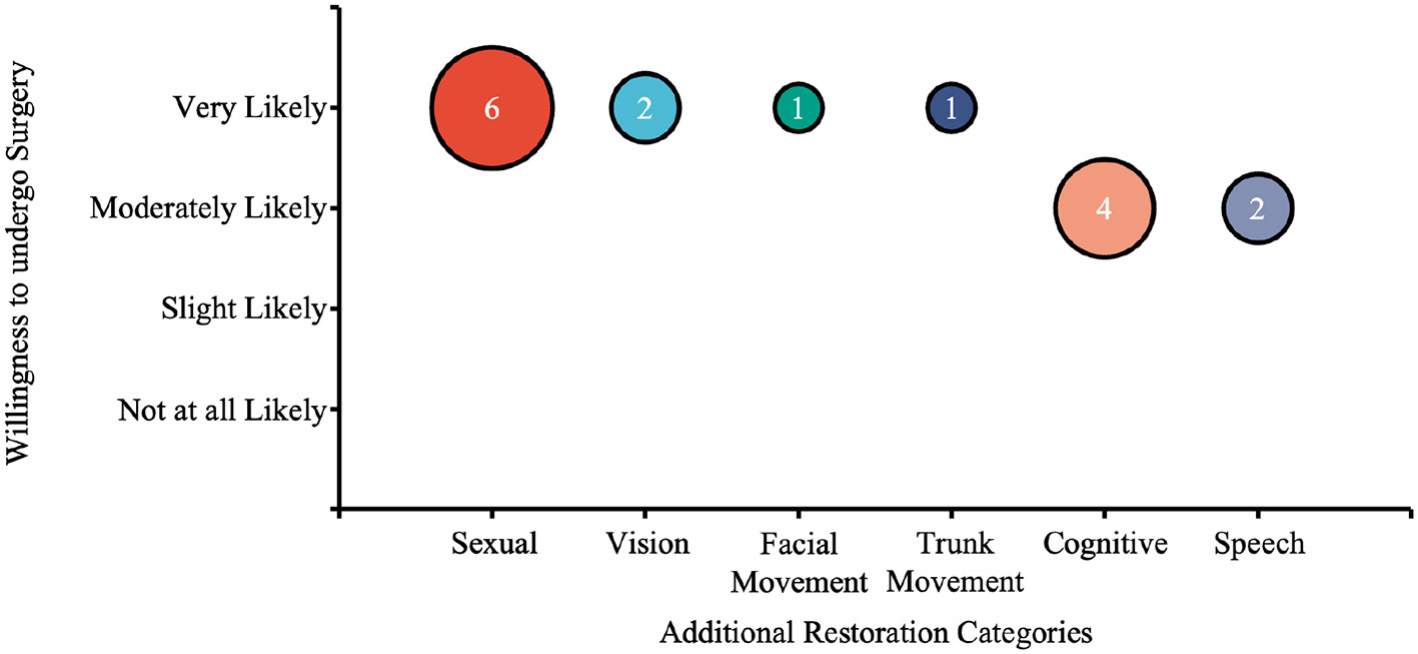
A bubble chart illustrating willingness to undergo surgery for additional restoration categories. The bubble size represents the number of participants interested in each category, and the categories are ordered by average likelihood of undergoing surgery. The number in the bubble represents the number of answers. Willingness was rated on a scale from “Not at all Likely” to “Very Likely,” with an average likelihood calculated for each category. sexual restoration had the highest interest, followed by vision, facial movement, trunk movement, cognitive function, and speech.

In Part 4, the additional restorations indicated by participants were categorized and ranked based on their average willingness to undergo surgery for a given function (Figure 11). The most frequently endorsed category was sexual function, with six participants indicating they were “very Likely” on average to undergo surgery for its restoration. Other notable categories in descending order of interest are vision, facial movement, trunk movement, cognitive function, and speech. Representative participant responses regarding additional desired functions included: “sexual sensations, the ability to get erections,” “field of vision,” and “increased symmetry in the face,” “unknown side effects and/or long term effects of the device,” “brain damage from surgery,” and “no guarantee of functional restoration.”

### 3.9 Concerns

**Figure 12:**
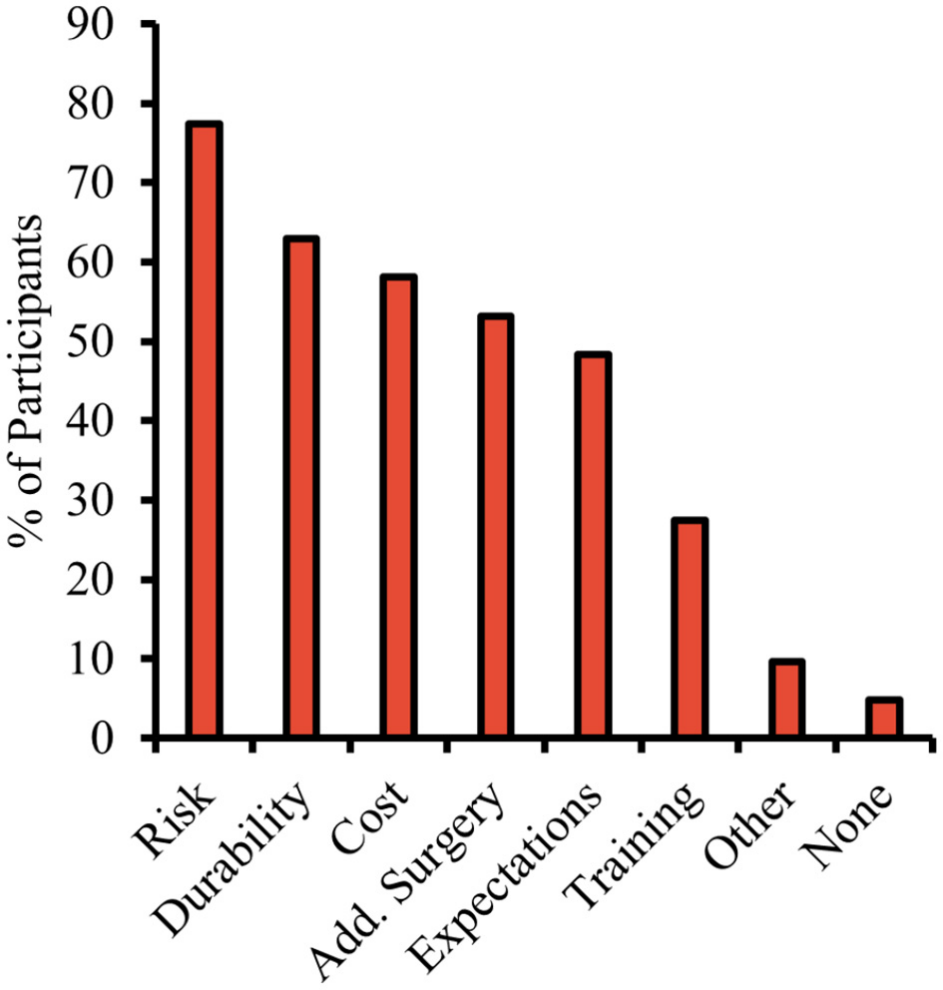
Rank ordered categories of concerns regarding undergoing prospective ECoG-based BCI implantation. Participants were highly concerned about surgical risk and potential complications. Other concerns included cost, the need for additional surgeries, failure of implanted BCI to meet practical expectations, time needed for BCI to be trained, etc. When asked to elaborate on concerns, participants noted: “unknown side effects and/or long term effects of the device,” “brain damage from surgery,” and “no guarantee” of functional restoration.

In part 4, broad categories of concerns raised by the participants are rank ordered and summarized in Figure 12. Potential risks and possible complications of surgery, such as infection, scarring, excessive bleeding, blood clots, and reactions to anesthesia, were the most commonly cited concern (77%). Next, 62% raised concerns about long-term usage and durability of device.

## 4 Discussion

This study examined the receptiveness of stroke and SCI cohorts to undergoing surgery to implant ECoG-based BCIs and how it may have been influenced by rehabilitation priorities and prospective level of functional recovery. The results indicated that the stroke population may focus more on upper extremity restoration over lower extremity restoration, and vice versa for the SCI population. The vast majority of stroke and SCI participants were very or moderately willing to undergo surgical implantation of an ECoG-based BCI to restore a given upper/lower extremity motor/sensory as well as autonomic functions. Willingness to undergo surgery was not influenced by the promise of more restorative features within the motor and sensory domains. Disability severity also did not affect willingness to undergo an implantation of a BCI device. Similarly, willingness to undergo surgery was not affected by prior knowledge of BCI technology, with the only exception that stroke participants with prior BCI knowledge were more willing to undergo surgical BCI implantation if it could restore grasp/release finger and arm control. Participants indicated that major concerns for such a surgery included potential surgical risks (e.g., infection, bleeding, and anesthesia complications), the need for additional surgeries if the BCI fails, concerns about long-term durability and reliability of the device, high cost, extensive training time, and the possibility that the BCI may not meet functional expectations.

Functional priorities found in this cohort are consistent with prior literature reports on rehabilitation priorities in the SCI and stroke population (Lam et al., 2022; Lo et al., 2016). Specifically, the stroke survivor population likely places more emphasis on upper extremities given that many (70%) are chronically affected by significant upper extremity impairment (Dutta et al., 2022), whereas ~80% are ambulatory in the chronic phase (Eng and Tang, 2007; Purton et al., 2023). As such, it is not surprising that restoration of upper extremity function receives more attention. Conversely, chronic SCI participants who have long-term disabilities are almost universally affected by gait impairment, with 73.3% of participants reporting being wheelchair dependent (Table 2, excluding “Not applicable”). This likely explains why functional restoration of the lower extremities is seen as a higher priority in a larger proportion of SCI participants compared to upper extremity or sensation functions.

It was unexpected that willingness to undergo surgery for implantation of the ECoG grid and BCI device was not clearly associated with the extent of disability, the perceived importance of functional restoration, or prior knowledge of BCIs. Furthermore, contrary to our expectations, participants did not express greater willingness to undergo surgery when more functions could potentially be restored. Instead, there appeared to be consistent willingness to consider implantation of ECoG-based BCIs for functional restoration in general. Given the novelty and perceived promise of BCI technology, it is crucial to consider the ethical implications. Patients with severe disabilities may be especially susceptible to optimistic portrayals of emerging neurotechnologies, potentially leading to premature or inadequately informed consent. Clear clinical guidelines and robust consent frameworks will be essential to safeguard against the risk of unnecessary surgical interventions driven by technological optimism rather than established benefit.

A notable exception to this is that prior knowledge played a role in willingness to undergo BCI implantation surgery for upper extremity motor function restoration amongst stroke participants. It is possible that stroke survivors are more likely to actively search for means to restore upper extremity function, and therefore have likely been exposed to BCI. In turn, this may have galvanized them toward acceptance of BCI technology. Nevertheless, the overarching observation suggests to BCI developers that the first implantable BCI products may not have to provide drastic levels of complexity to gain acceptance with potential recipients. Similarly, BCI developers can also expect that the potential market may not necessarily only involve participants with severe disability. For example, although not explicitly studied here, it may be inferred that participants with even moderate disability would still be willing to undergo surgery for BCI systems that could improve neurological functions. This is opposed to simply being used as a neuroprosthetic for participants with complete loss of neurological function.

Whereas it was previously unknown how sensory restoration via BCI technology would be received among the SCI and stroke survivor populations, this study found that interest in the restoration of somatic sensation in the upper and lower extremities is high. This outcome suggests that invasive ECoG-BCI systems that offer bi-directional features may also be of significant interest.

Amongst those affected by bladder or bowel function and sensation, there was a very high level of interest in implantable ECoG-based BCI systems that can hypothetically restore such autonomic functions. Similar to prior studies (Anderson, 2004; Ditunno et al., 2008), bladder and bowel functions have often been rated as equal to or higher in priority than upper or lower extremity motor functions, particularly among individuals with high-level spinal cord injuries. With very limited treatment options for neurogenic bladder and bowel following stroke and SCI, these results suggest that BCI systems seeking to address these issues would be well received. Emerging work exploring the integration of BCIs with sacral nerve stimulation for closed-loop bladder control (Aamir and Siddiqui, 2025) represents a promising direction, though this area remains largely underexplored and merits significant research effort.

*Post-hoc* demographic analyses revealed that higher household income was significantly associated with greater willingness to undergo surgery for sensory restoration specifically (Table 3), but not for motor or autonomic restoration (Supplementary Table 1). These findings suggest that individuals with higher income might have a higher interest in invasive BCI technology, particularly if sensory restoration is involved. The absence of significant associations with age, gender, or education level (Supplementary Table 1) indicates that interest in BCI technology is broadly distributed across the target population, though equitable access considerations will be important as these technologies approach clinical availability.

While our survey specifically described an ECoG-based BCI system, the findings regarding patient interest and functional priorities may reasonably extend to other implantable BCI modalities, including MEA-based and endovascular approaches. We expect that most potential recipients are unlikely to distinguish between electrode implant types when considering surgical intervention for functional restoration.

### 4.1 Limitations

Several limitations warrant consideration. Our participants were recruited from UC Irvine clinics and rehabilitation support networks, which likely captured individuals actively engaged in their recovery, and may lead to participant bias. While this may limit generalizability to the general public, our work still provides valuable initial insight into the attitudes to significant segments of the target population. The modest sample size led to small subgroup sizes, which limited statistical power for those comparisons and may have contributed to non-significant findings. However, the results still provide preliminary data that can inform the design of a larger study, particularly one that includes a more general target population as above.

As the key variables are both new to this survey and not combined into scales with other items, they offer no clear comparisons to test against for reliability. However, a comprehension pretest may have helped clarify the validity of these measures by assessing in qualitative interviews how well target participants comprehended the terminology and questions. Without such testing, it is possible that some participants misunderstood questions, particularly about BCI treatment options.

Another limitation is that we cannot predict how well self-reported willingness to consider hypothetical BCI treatments would correlate with actual consent decisions; a study to test that could not be conducted until BCI is available for clinical use and would require a larger sample and a longitudinal design. The gap between patients' health-related intentions and their achieved behaviors has been extensively studied, so we would expect far lower uptake than implied here, yet those who intend a behavior are more likely to pursue it than those who do not (Jekauc et al., 2025). Survey-based assessment of emerging technologies is a well-established approach for gauging patient priorities prior to clinical availability, and the consistent willingness across injury severities and functional categories suggests genuine underlying interest.

A significant number of participants (64%) did not respond to the mobility aid question (Question 7); the reason for this high non-response rate is unclear but may reflect question ambiguity or technical issues. Among those who did respond, the wheelchair reliance rates in our sample (73.3% for SCI, 27.3% for stroke) are consistent with published literature (Mountain et al., 2010; Ekiz et al., 2014), supporting the representativeness of our cohort among actively rehabilitating individuals.

### 4.2 Ethical considerations

Given the novelty and perceived promise of BCI technology, it is crucial to consider the ethical implications of the level of interest observed in this study. Patients with severe disabilities may be especially susceptible to optimistic portrayals of emerging neurotechnologies, potentially leading to premature or inadequately informed consent (Yuste et al., 2017). The finding that willingness to undergo surgery was not clearly associated with disability severity or the extent of functional restoration offered raises concerns about whether participants are making fully informed risk-benefit assessments. Clear clinical guidelines and robust consent frameworks will be essential to safeguard against the risk of unnecessary surgical interventions driven by technological optimism rather than established benefit. Ongoing efforts to develop neuroethics frameworks for neurotechnology (Yuste et al., 2017) provide important guidance as the field moves toward clinical translation.

### 4.3 Future directions

Several directions for future research emerge from this study. First, longitudinal studies are needed to track how attitudes toward BCI technology evolve over time, particularly as participants learn more about the technology or as BCIs become more clinically available. Second, cross-cultural studies would help assess the generalizability of our findings to diverse populations with different healthcare systems, cultural attitudes toward technology, and disability perspectives. Third, recruiting a larger cohort from the general SCI and stroke population (not limited to those actively engaged in rehabilitation clinics, and with a priori power calculations based on the current results) would improve generalizability and provide sufficient statistical power for demographic subgroup analyses. Such studies should conduct extensive comprehension pretesting as well as any other relevant validation of measures. Fourth, studies correlating expressed interest with actual consent decisions in clinical trials would provide important validation of survey-based findings. Finally, qualitative research exploring the reasoning behind participants' willingness (or reluctance) would provide richer context for understanding these attitudes.

### 4.4 Conclusions

In summary, the majority of both stroke and SCI participants in this actively rehabilitating cohort expressed a high willingness to undergo surgery for ECoG-based BCI implantation, regardless of the extent of their disability or the specific level of function offered by the device. While these findings should be interpreted cautiously given the hypothetical nature of the survey and potential participation bias, they suggest that even relatively simple or narrowly focused implantable BCI systems may find receptive users among motivated individuals with neurological injuries. Notably, bi-directional BCIs (BDBCI) that can restore both motor and sensory functions—particularly grasp and somatic sensation—were of substantial interest, especially among stroke survivors with prior BCI knowledge. Additionally, systems targeting autonomic functions, such as bladder and bowel control, generated strong interest across both populations, reaffirming findings from prior rehabilitation surveys and highlighting a critical unmet need.

These results suggest considerable interest for clinically viable, implantable BCI systems among actively engaged SCI and stroke populations, particularly for systems that address motor, sensory, and autonomic deficits. Notably, current commercial BCI efforts (e.g., Neuralink, Synchron) have primarily focused on communication restoration for individuals with ALS or severe dysarthria or anarthria. Our findings suggest that motor, sensory, and autonomic restoration for SCI and stroke populations represent substantial unmet needs that may warrant greater attention from BCI developers, potentially representing a larger cohort of interested recipients than communication-focused applications alone. However, this interest must be balanced against the ethical imperative to ensure that potential recipients make fully informed decisions. This concern aligns with emerging neuroethics frameworks that emphasize cognitive liberty, mental privacy, and protection of vulnerable populations from exploitation in neurotechnology research (Yuste et al., 2017). As the BCI field advances toward clinical deployment, the development of robust consent frameworks and realistic expectation-setting will be as important as technological innovation itself.

## Data Availability

The raw data supporting the conclusions of this article will be made available by the authors, upon request without undue reservation.
