# Supplementary material for: Perception of brain-computer interface implantation surgery for motor, sensory, and autonomic restoration in spinal cord injury and stroke
Source: Front Neurosci. 2026 Mar 18;20:1678175. doi: 10.3389/fnins.2026.1678175 (PMC13039023; doi:10.3389/fnins.2026.1678175)
Supplement: Supplementary file 1 [file Data_Sheet_1.pdf]

Supplementary Material

1 SURVEY

Top of Form

University of California, Irvine

Study Information Sheet

Functional Priorities of Stroke and Spinal Cord Injury Patients for Invasive Procedures

Lead Researcher

Tracie Tran, Jr. Specialist, UC Irvine BCI Lab

Neurology

traciet@uci.edu

Faculty Sponsor

An Do, M.D., Assistant Professor

Neurology

and@uci.edu

Other Researchers

Zoran Nenadic, Ph.D., Assistant Professor

Biomedical Engineering

znenadic@uci.edu

Gabrielle Matias, Jr. Specialist, UC Irvine BCI Lab

Neurology

matiasje@uci.edu

- You are being asked to participate in a research study that is intended to help us understand your priorities and concerns for future research in emerging technologies that are capable of restoring movement and sensation after spinal cord injury or stroke. We hope to use your insights to improve future studies in this field by accommodating your needs and expectations.
- You are eligible to participate in this study if you 18 years of age or older and suffer from motor impairment due to stroke or spinal cord injury.
- The research procedures involve an online anonymous survey that is estimated to take approximately 10 minutes to complete. Upon completion, you will receive a $10 Amazon gift card as a token of appreciation for your time.

- Possible risks/discomforts associated with the study include a potential breach of confidentiality, although the utmost care will be taken to avoid this.
- There are no direct benefits from participation in the study. However, this study may yield insights into current concerns and priorities of stroke and spinal cord injury populations that may guide future brain computer interface research.
- All research data collected will be stored securely and confidentially. No personal identifiers will be collected or stored as the survey will remain anonymous.
- The research team, authorized UCI personnel, and regulatory entities, may have access to your study records to protect your safety and welfare. Any information derived from this research project that personally identifies you will not be voluntarily released or disclosed by these entities without your separate consent, except as specifically required by law.
- If you have any comments, concerns, or questions regarding the conduct of this research please contact the researchers listed at the top of this form.
- Please contact UCI's Office of Research by phone, (949) 824-6662, by e-mail at IRB@research.uci.edu or at 5171 California Avenue, Suite 150, Irvine, CA 92697 if you are unable to reach the researchers listed at the top of the form and have general questions; have concerns or complaints about the research; have questions about your rights as a research subject; or have general comments or suggestions.
- Participation in this study is voluntary. There is no cost to you for participating. You may choose to skip a question or a study procedure. You may refuse to participate or discontinue your involvement at any time without penalty. You are free to withdraw from this study at any time. If you decide to withdraw from this study you should notify the research team immediately.
- As this survey is anonymous, the study team may not be able to extract or delete any specific data provided, should the subject choose to withdraw from the study.
- As part of the completion of this survey, you are agreeing to the "Terms of Use" of eSurv.org, the entity administering the survey. The data you provide may be collected and used by this agent, as per its privacy agreement. Note: There is no reasonable expectation that data is anonymous.

By clicking "Next," you are consenting to partake in the survey.

---

1. Please select the type of injury you have.

☐Spinal Cord Injury
☐Stroke
☐Don't know/Decline to Answer

---

2. If known, what is your type of injury?

☐Tetraplegia (Unable to move/feel both arms and legs)
☐Paraplegia (Unable to move/feel lower half of body)
☐I do not know/Decline to Answer

3. Please indicate your level of injury on the ASIA Impairment Scale *(if known)*.

☐Grade A: Complete: No sensory or motor function is preserved in the sacral segments S4-S5.

☐Grade B: Incomplete: Sensory but not motor function is preserved below the neurological level and includes the sacral segments S4-S5.
☐Grade C: Incomplete: Motor function is preserved below the neurological level, and more than half of the key muscles below the neurological level have a muscle grade less than 3.
☐Grade D: Incomplete: Motor function is preserved below the neurological level, and at least half of the key muscles below the neurological level have a muscle grade greater than or equal to 3.
☐Grade E: Motor and sensory functions are normal
☐I do not know/Decline to answer

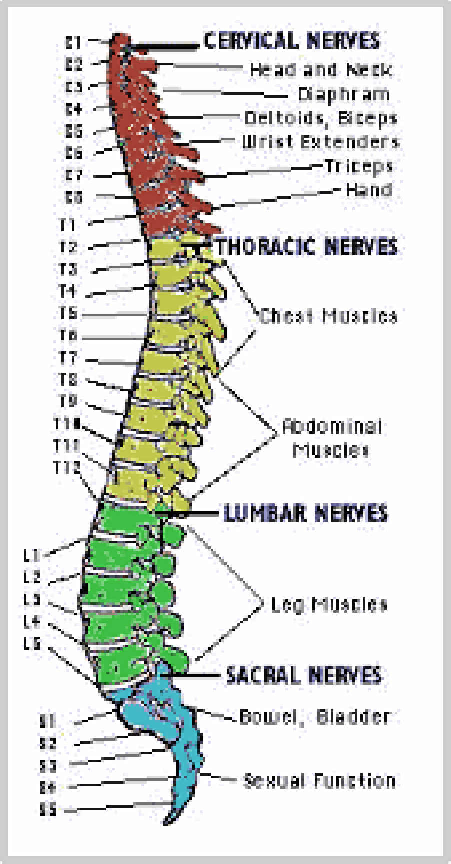

4. If known, what is your level of impairment?

☐C1
☐C2
☐C3
☐C4
☐C5
☐C6
☐C7
☐C8
☐T1
☐T2
☐T3
☐T4
☐T5

☐T6
☐T7
☐T8
☐T9
☐T10
☐T11
☐T12
☐L1
☐L2
☐L3
☐L4
☐L5
☐S1
☐S2
☐S3
☐S4-5
☐I do not know/Decline to Answer

---

5. Please indicate your current level of disability on the scale below. *Please select the description that best describes you.*

☐0 - No symptoms
☐1 - No significant disability. Able to carry out all usual activities, despite some symptoms.
☐2 - Slight disability. Able to look after own affairs without assistance, but unable to carry out all previous activities.
☐3 - Moderate disability. Requires some help, but able to walk unassisted.
☐4 - Moderately severe disability. Unable to attend to own bodily needs without assistance, and unable to walk unassisted.
☐5 - Severe disability. Requires constant nursing care and attention, bedridden, incontinent.
☐I do not know/Decline to Answer

---

6. Please indicate your current type of walking aid.

☐I use a walking aid such as a cane or leg brace
☐I use a wheelchair
☐Other

The next few questions are about your opinions on brain-computer interfaces.

7. Have you heard of brain-computer interfaces prior to this survey?

☐Yes
☐No

Brain-computer interfaces seek to restore movement or sensation by connecting a person's brain to an external device. This allows a previous motor function or sensation to be restored by bypassing the site of injury and recovering thought control. For example, brain computer interfaces can allow a person to move a robotic arm or regain the sensation of bladder fullness. In the future, we hope to develop a system that is fully implantable.

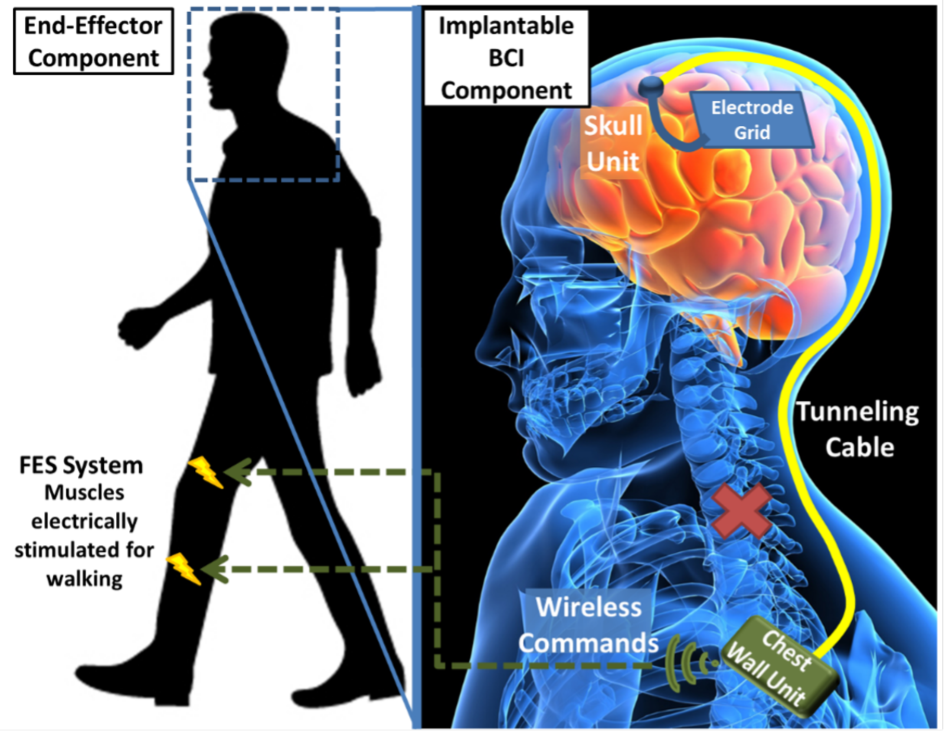

8. How likely are you to consider surgery for a fully implantable brain-computer interface system that would be approximately the size of a pacemaker?

☐Very likely

☐Moderately likely

☐Slightly likely

☐Not at all likely

In order for brain-computer interfaces to work, brain waves must be constantly recorded. One of the current methods to do this is electrocorticography (ECoG). In ECoG, an electrode grid is placed directly on the surface of the brain to record activity from which a person's intentions can be deduced. For this procedure, surgery is required to permanently implant the grid under the skull.

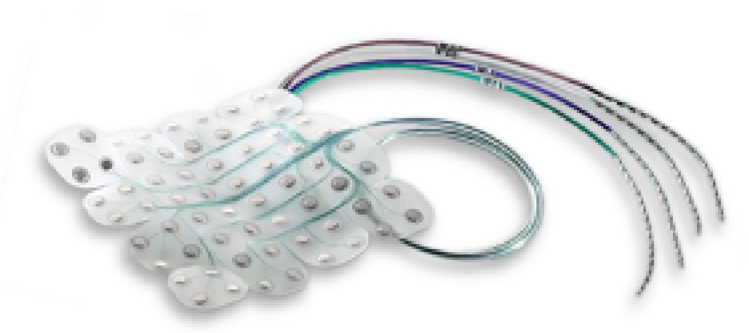

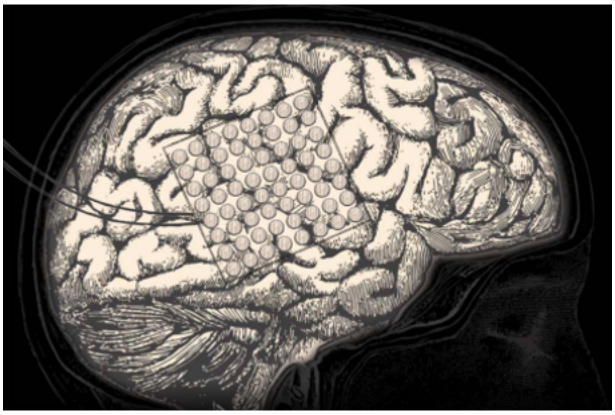

In the next few questions, we are interested in learning whether or not you would be willing to undergo procedures for ECoG implantation if certain motor and sensory functions can be restored.

9. Please indicate how important regaining arm or upper body function is to you.

☐Very Important

☐Moderately Important

☐Slightly Important

☐Not At All Important

☐Not Applicable

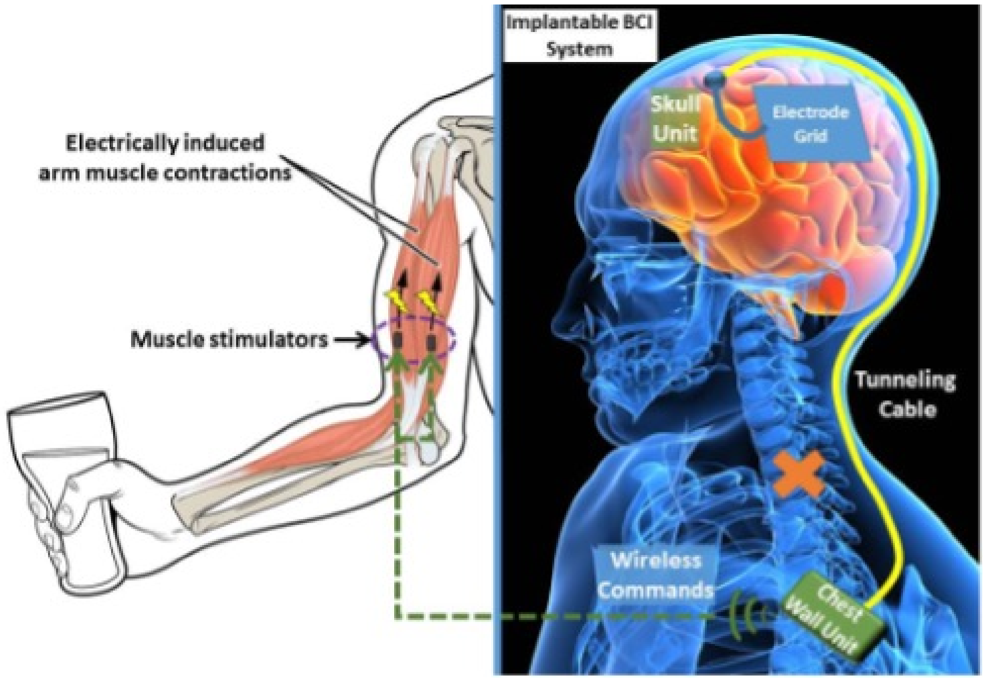

In this setup, an ECoG electrode grid is implanted surgically in the brain to record electrical activity. The signal then gets sent to a chest wall unit where the person's intentions are decoded and translated into commands that are sent wirelessly to implanted muscle stimulators in the arm. The muscles in the arm can therefore be induced to contract and make movements that this person wanted to make.

A similar setup can also be done with a robotic arm, as shown.

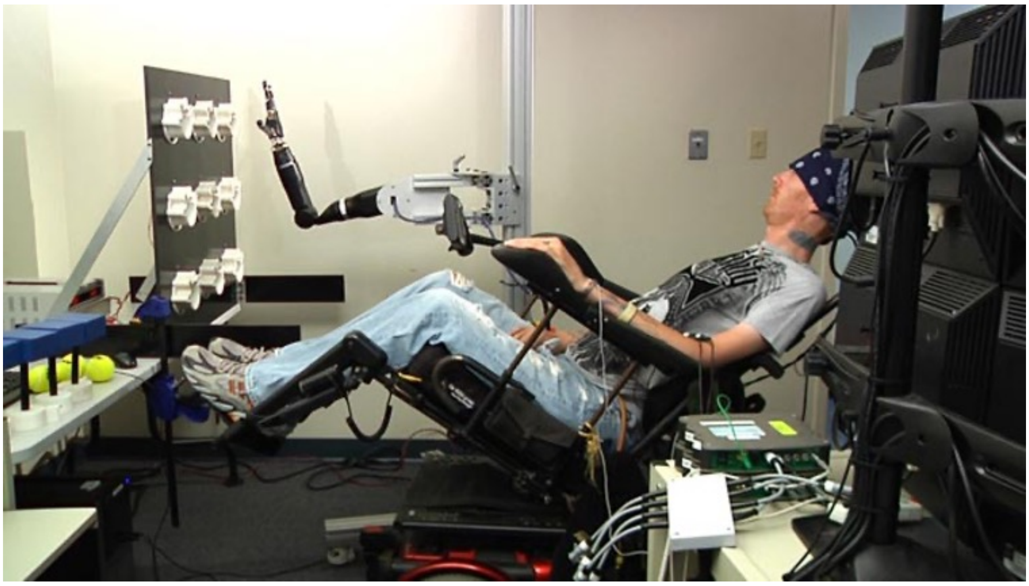

YouTube, uploaded by UPMC, 07 October 2011, https://www.youtube.com/watch?v=yff20TlHv34, Permissions: YouTube Terms of Service

10. How likely would you consider surgery to implant an electrode grid if basic grasp/release ability can be restored?

☐Very likely

☐Moderately likely

☐Slightly likely

☐Not at all likely

☐Not Applicable/Decline to Answer

11. How likely would you consider surgery to implant an electrode grid if fine control of fingers, in addition to basic grasp/release ability, can be restored?

☐Very likely

☐Moderately likely

☐Slightly likely

☐Not at all likely

☐Not Applicable/Decline to Answer

12. How likely would you consider surgery to implant an electrode grid if fine control of your arm, fine control of your fingers, and basic grasp/release ability can all be restored?

☐Very likely

☐Moderately likely

☐Slightly likely

☐Not at all likely

☐Not Applicable/Decline to Answer

---

13. Please indicate how important regaining the ability to walk is to you.

☐Very Important

☐Moderately Important

☐Slightly Important

☐Not At All Important

☐Not Applicable

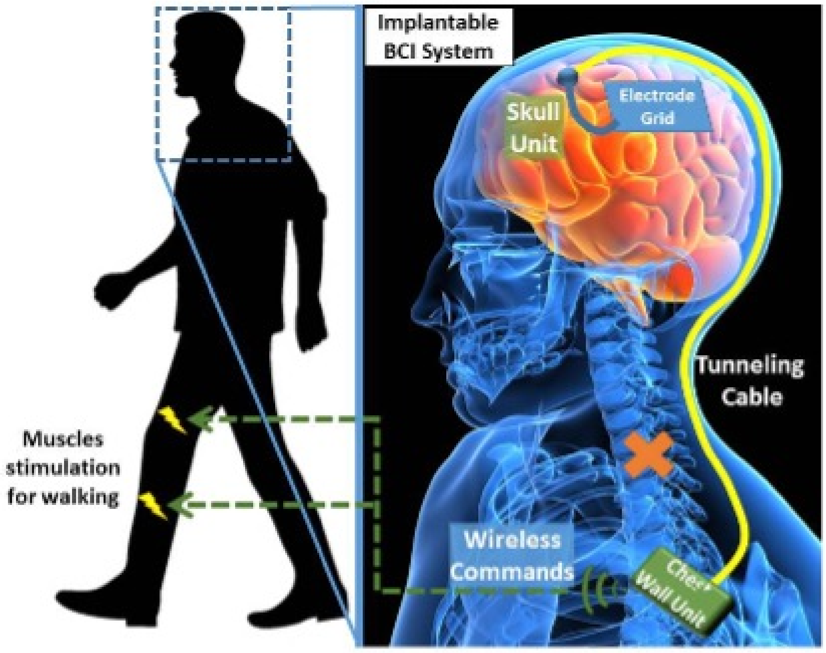

In this setup, an ECoG electrode grid is implanted surgically in the brain to record electrical activity. The signal then gets sent to a chest wall unit where the person's intentions are decoded and translated into commands that are sent wirelessly to implanted muscle stimulators in the leg. The leg muscles can therefore be controlled to make stepping movements for walking.

14. How likely would you consider surgery to implant an electrode grid if you can regain the ability to stand?

☐Very likely
☐Moderately likely
☐Slightly likely
☐Not at all likely
☐Not Applicable/Decline to Answer

15. How likely would you consider surgery to implant an electrode grid if you can regain the ability to walk at a constant speed?

☐Very likely
☐Moderately likely
☐Slightly likely
☐Not at all likely
☐Not Applicable/Decline to Answer

16. How likely would you consider surgery to implant an electrode grid if you can control walking at various speeds?

☐Very likely
☐Moderately likely

☐Slightly likely
☐Not at all likely
☐Not Applicable/Decline to Answer

17. How likely would you consider surgery to implant an electrode grid if you can make turns in addition to being able to control walking at various speeds?

☐Very likely
☐Moderately likely
☐Slightly likely
☐Not at all likely
☐Not Applicable/Decline to Answer

---

18. Please indicate how important regaining sensation is to you.

☐Very Important
☐Moderately Important
☐Slightly Important
☐Not At All Important
☐Not Applicable/Decline to Answer

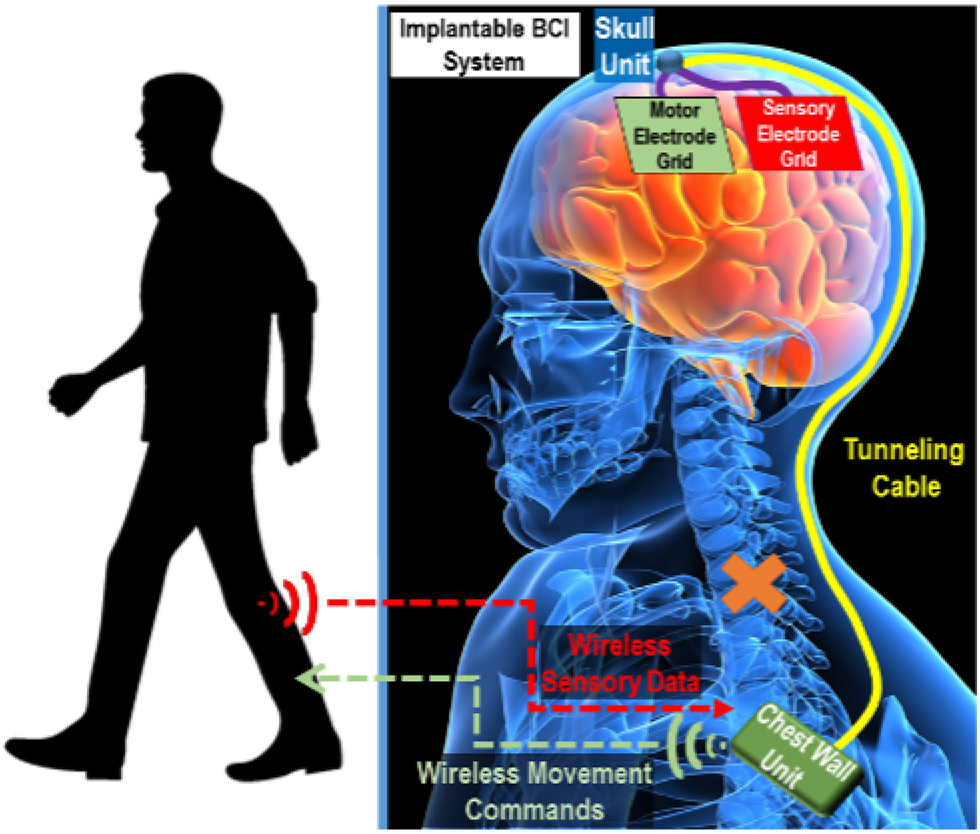

In this set-up, an ECoG electrode grid is implanted surgically in the brain to record electrical activity. The signal then gets sent to a chest wall unit where the person's intentions are decoded and translated

into commands that are sent wirelessly to implanted muscle stimulators in the leg. The leg muscles can therefore be controlled to make stepping motions for walking.

In addition, sensors placed in the leg can detect when legs are moving and when the feet are placed on the ground. This information is sent to the chest wall unit and converted to signals for the sensory areas of the brain. These signals can provide sensation of leg movements to a person who has lost leg sensation.

Similar set-ups can be used to regain sensation in other parts of the body.

19. How likely would you consider surgery to implant an electrode grid if sensation in your legs can be restored?

☐Very likely

☐Moderately likely

☐Slightly likely

☐Not at all likely

☐Not Applicable/Decline to Answer

20. How likely would you consider surgery to implant an electrode grid if sensation in your arms can be restored?

☐Very likely

☐Moderately likely

☐Slightly likely

☐Not at all likely

☐Not Applicable/Decline to Answer

21. How likely would you consider surgery to implant an electrode grid if sensation in your hands and fingers can be restored?

☐Very likely

☐Moderately likely

☐Slightly likely

☐Not at all likely

☐Not Applicable/Decline to Answer

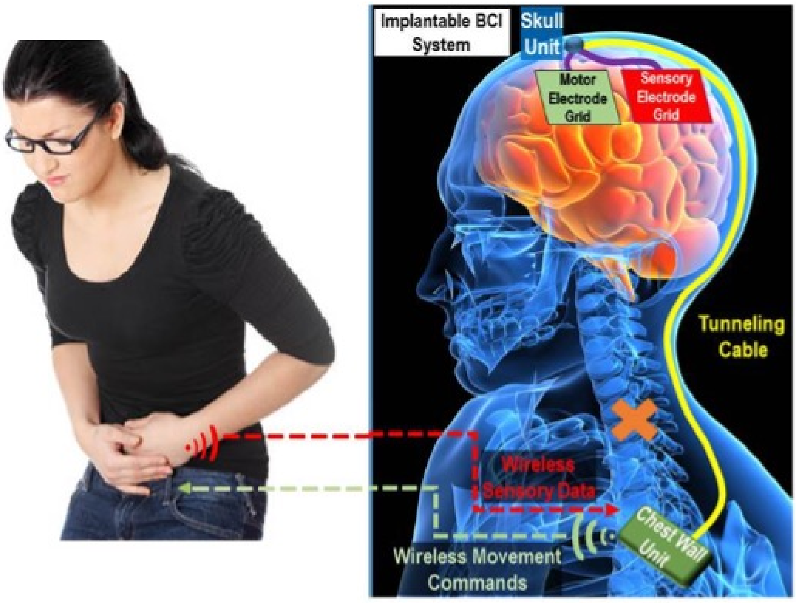

Adapted with permission from DespositPhotos, under standard license (https://depositphotos.com/photo/young-woman-stomach-issues-isolated-white-4965208.html).

In this set-up, an ECoG electrode grid is implanted surgically in the brain to record electrical activity. The signal then gets sent to a chest wall unit where the person's intentions are decoded and translated into commands that are sent wirelessly to implanted muscle stimulators in the sphincters that control urination and defecation. The person can therefore control when they would like to use the restroom.

In addition, sensors placed on the bladder can detect when the bladder is full. This information is sent to the chest wall unit and converted to signals for the sensory areas of the brain. These signals can provide the sensation of bladder fullness to a person who has lost this ability.

22. How likely would you consider surgery to implant an electrode grid if you can regain the sensation of bladder fullness?

☐Very likely
☐Moderately likely
☐Slightly likely
☐Not at all likely
☐Not Applicable/Decline to Answer

23. How likely would you consider surgery to implant an electrode grid if you can control when you use the bathroom?

☐Very likely
☐Moderately likely
☐Slightly likely
☐Not at all likely
☐Not Applicable/Decline to Answer

24. Is there any other bodily function you would like to restore that has not been mentioned here?

☐Yes
☐No

25. Please explain what motor function(s) or sensation you would like to restore.

Text box

26. How likely would you consider surgery to implant electrodes in the brain if the function you indicated can be restored?

☐Very likely
☐Moderately likely
☐Slightly likely
☐Not at all likely

27. Can you please explain why you would not be interested in surgery for electrode implantation in the brain if the desired function can be restored?

Text box

28. What kind of concerns would you have regarding a surgery to implant a BCI system? Select all answers that apply.

☐Cost of surgery for BCI implantation
☐Potential risks and possible complications of surgery such as infection, scarring, excessive bleeding, blood clots, reactions to anesthetics
☐Motor and/or sensory restoration not meeting expectations for practical use
☐Length and effort of training required to fully maximize BCI usage
☐Possible need for additional surgical interventions if BCI fails
☐Long-term usage and durability of device
☐None
☐Other

29. (Optional) Please indicate any other concern you have about surgery for electrode implantation or BCI technology that is not listed above.

Text box

Finally, we would like to know more about you, to help compare your answers to those of other participants. Again, all of this information is confidential.

30. What is your age?

☐18-24 years old
☐25-34 years old
☐35-44 years old
☐45-54 years old
☐55-64 years old
☐65-74 years old
☐75 years or older
☐I do not know/Decline to Answer

31. I am:

☐Male
☐Female
☐Other
☐Decline to Answer

32. Please indicate the highest level of education you have completed:

☐Some high school or less
☐High School Diploma or equivalent
☐Some college
☐College graduate
☐Advanced Degree (Master's, JD, MD, etc.)
☐I do not know/Decline to Answer

33. Please indicate your current occupation setting:

☐Laborer or helper (examples: grounds maintenance worker, construction laborer)
☐Operative (examples: machine operator, parking lot attendant, bus driver)
☐Craft worker (examples: electrician, plumber, construction worker, painter)
☐Service worker (examples: cook, food preparation worker, custodian)
☐Security support (examples: police officer, security guard)
☐Commercial/sales support (examples: sales supervisor, cashier, travel agent)
☐Medical support (examples: medical assistant, healthcare worker)
☐Administrative support (examples: office manager, library technician, secretary, payroll clerk, accounting assistant)
☐Technician (examples: laboratory technician, LPN, diagnostic related technologist)
☐Professional (examples: instructor, engineer, scientist, physician, pharmacist, registered nurse, librarian, computer programmer, HR specialist, accountant, financial analyst, athletic coach)
☐Manager or official (executive officer, mid-level manager)
☐Other

☐Don’t know

34. What is your current living situation?

☐I live in a skilled nursing facility with health care staff providing full-time assistance
☐I live in an assisted living facility with health care staff providing partial assistance
☐I live at home, but use supportive living services (a health care worker provides assistance when needed)
☐I live at home, but somebody else (family, friend, caregiver) helps me complete daily tasks
☐I live at home and am able to complete daily tasks without assistance
☐Don’t know/Decline to answer

35. What is your household’s annual income? Include earnings, Social Security, disability payments, and any other type of income.

☐$9,999 or less
☐$10,000-$19,999
☐$20,000-$39,999
☐$40,000-$59,999
☐$60,000-$99,999
☐$100,000-$199,999
☐$200,000 or higher
☐I do not know/Decline to Answer

You have reached the end. Thank you for participating.

Bottom of Form

2 ADDITIONAL FIGURES

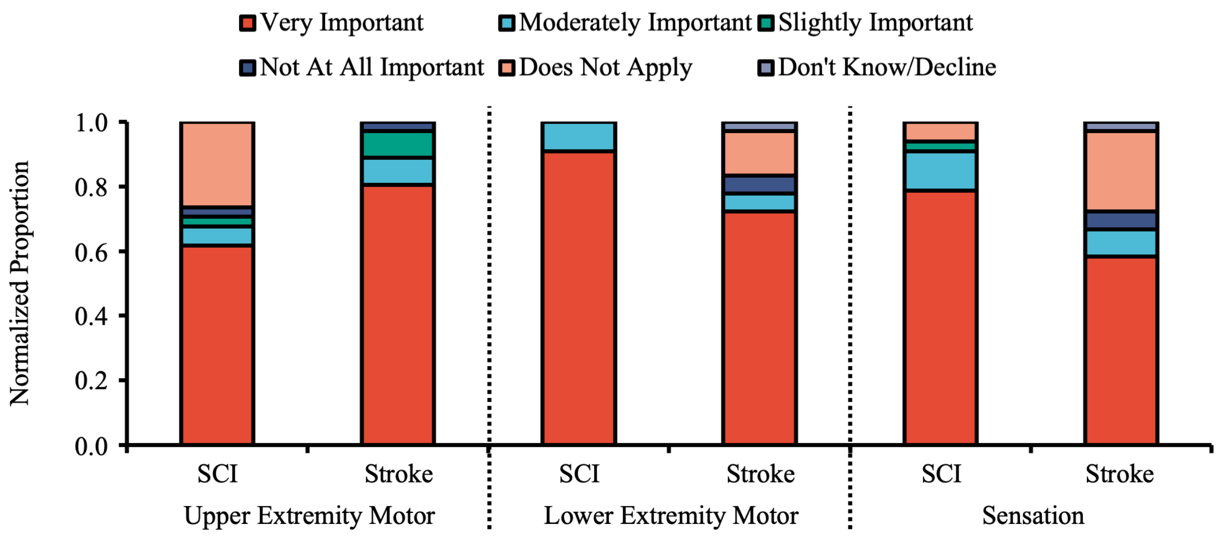

Figure a1: Importance of regaining motor and sensory functions to stroke and SCI participants as a normalized proportion. The majority ($> 60\%$) of both stroke and SCI participants indicated that regaining upper extremity motor function, lower extremity motor function, and extremities and bladder sensation were all "Very Important."

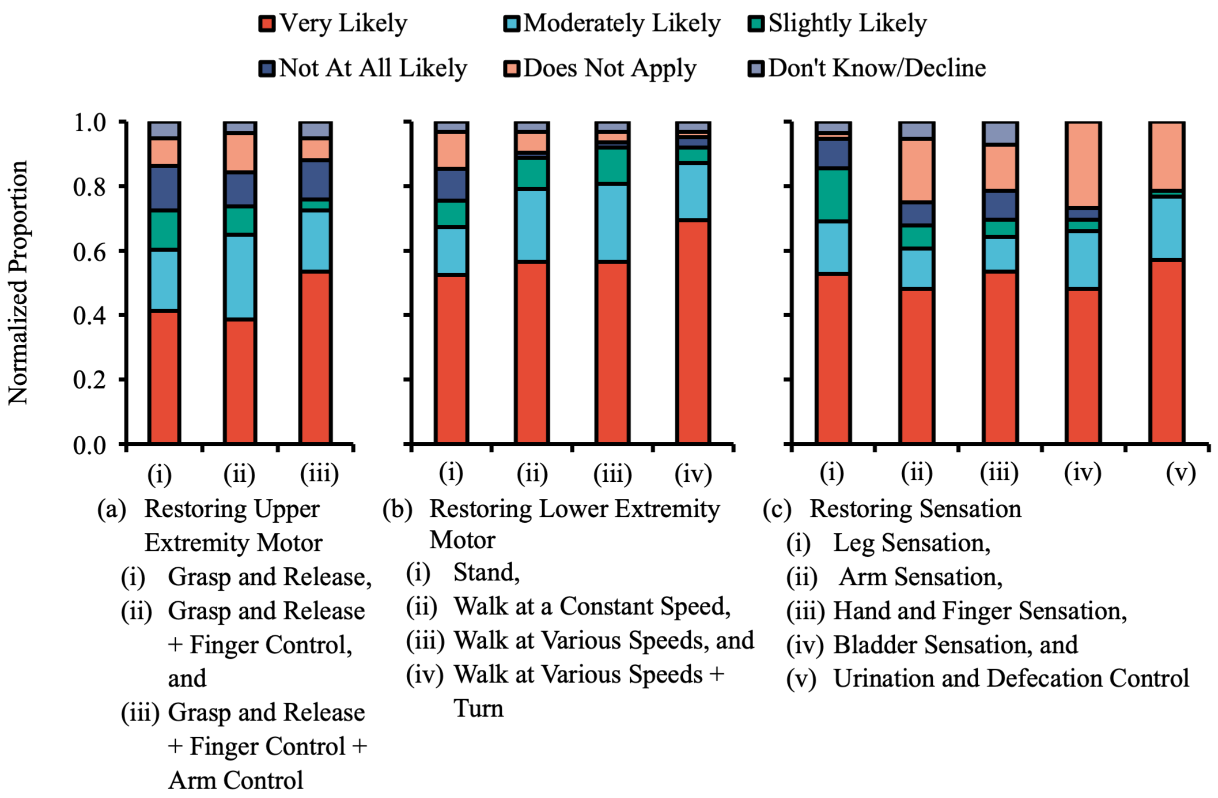

Figure a2: Participant willingness to undergo surgery to implant BCI system at various degrees of function restored. (a) Upper extremity motor function. (b) Lower extremity motor function. (c) Sensation. Participants were asked to indicate their level of disability as well as how important regaining upper extremity motor function, lower extremity motor function, and sensation were to them. Participants' responses were plotted as a sample size-normalized stacked bar graph of those who indicated that each function was "very important," "moderately important," "slightly important," "not at all important," or "does not apply." The majority of participants were at least moderately willing to undergo surgery to implant BCI systems for even basic levels of functional restoration.

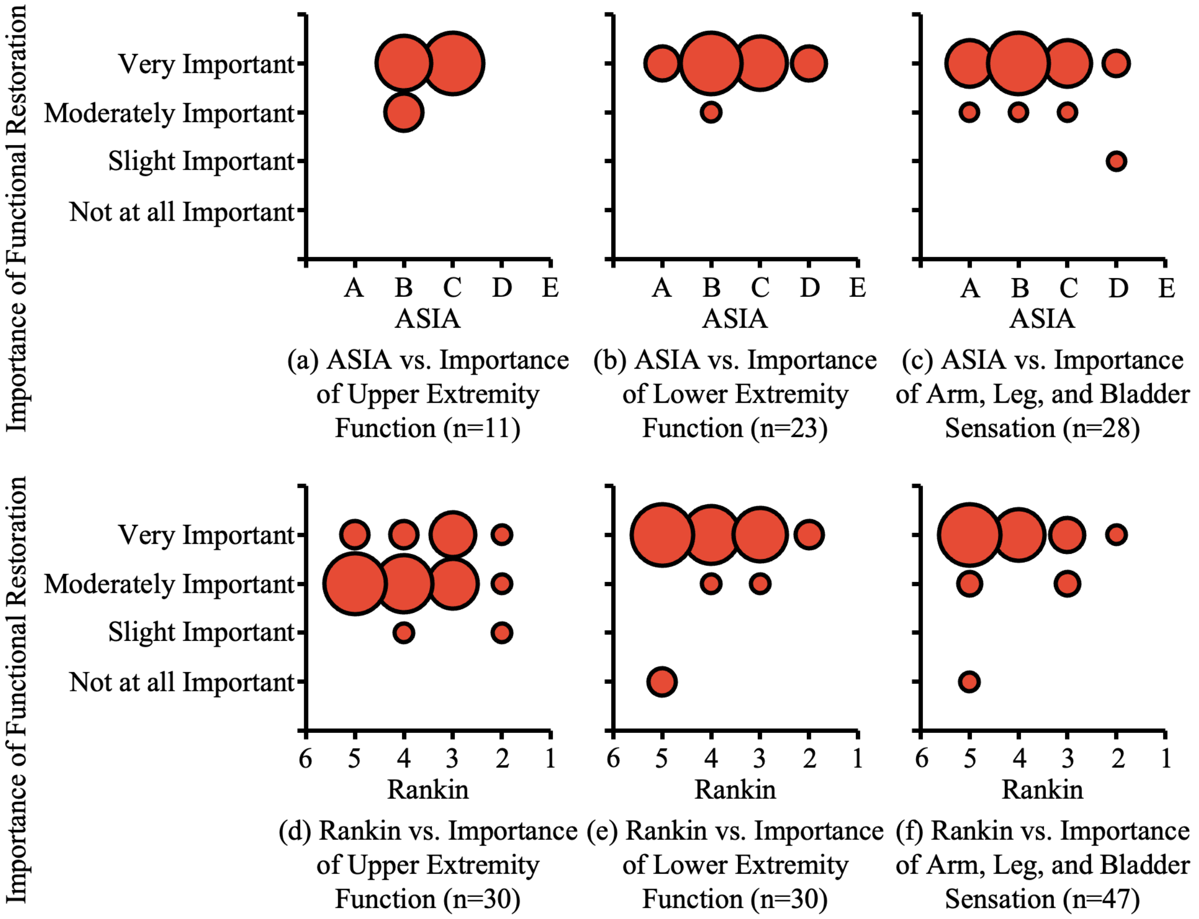

Figure a3: Importance of functional restoration given different levels of disability measured by ASIA Impairment or Modified Rankin Scale. ASIA scale ranks disability from A to E, with A being no motor, sensory or sacral sparing and E being normal motor and sensory function. Modified Rankin goes from 0 to 6 with 0 being no disability and 6 being dead. "Does not apply" responses were excluded.

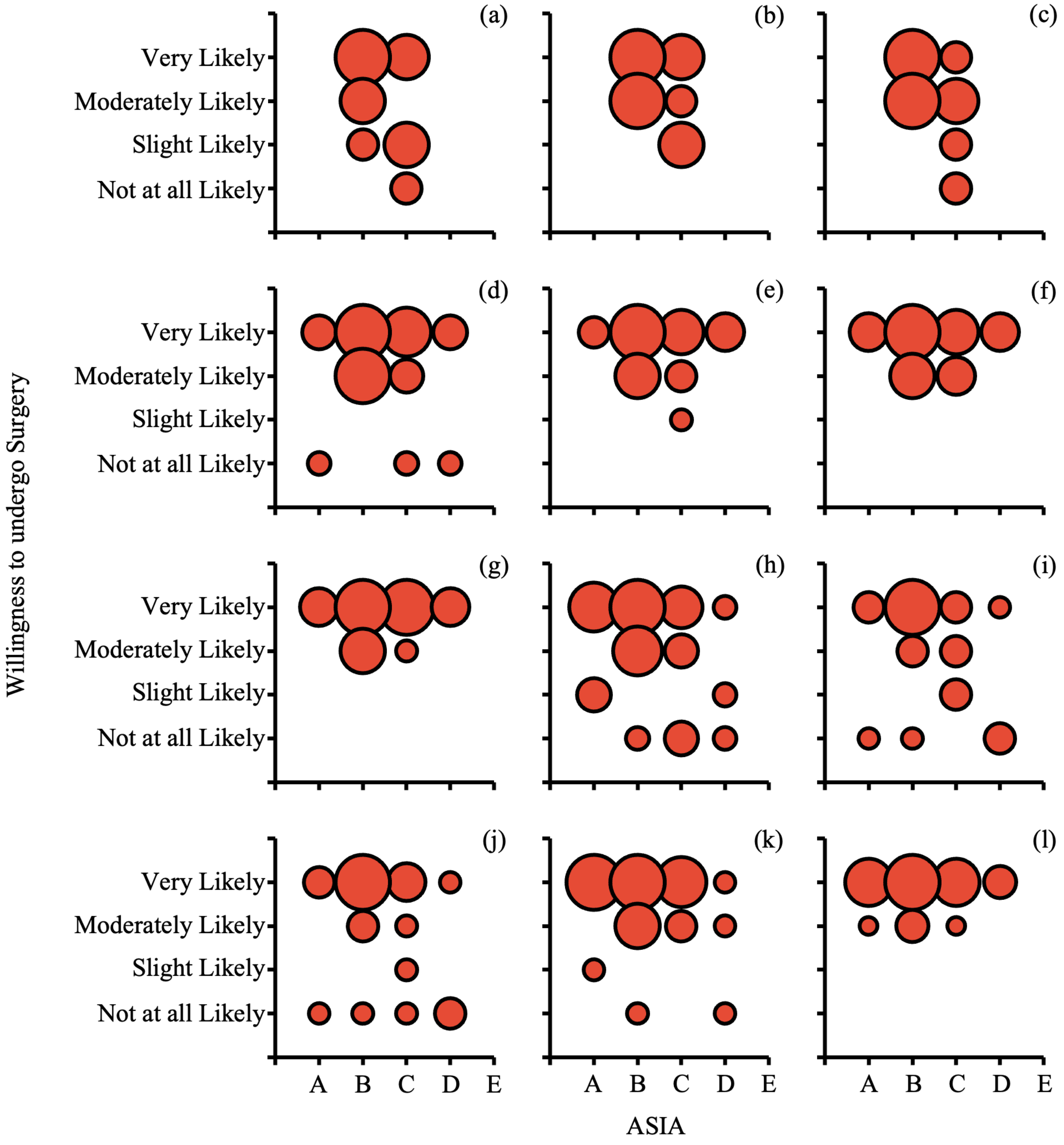

Figure a4: Participants' willingness to undergo surgery to restore different functions given different levels of disability measured by AISA Impairment Scale. ASIA scale ranks disability from A to E, with A being no motor, sensory or sacral sparing and E being normal motor and sensory function. "does not apply" was excluded from the bubble charts. ASIA vs. Restoring (a) Grasp and Release (n=11); (b) Grasp and Release + Finger Control (n=11); (c) Grasp and Release + Finger Control + Arm Control (n=11). ASIA vs. Restoring Ability to (d) Stand (n=23); (e) Walk at Constant Speed (n=22); (f) Walk at Various Speeds (n=23); (g) Walk at Various Speeds (f) + Turn (n=23); ASIA vs. Restoring (h) Leg Sensation (n=26); (i) Arm Sensation (n=21); (j) Hand and Finger Sensation (n=21); (k) Bladder Fullness Sensation (n=28); (l) Urination and Defecation Control (n=28);

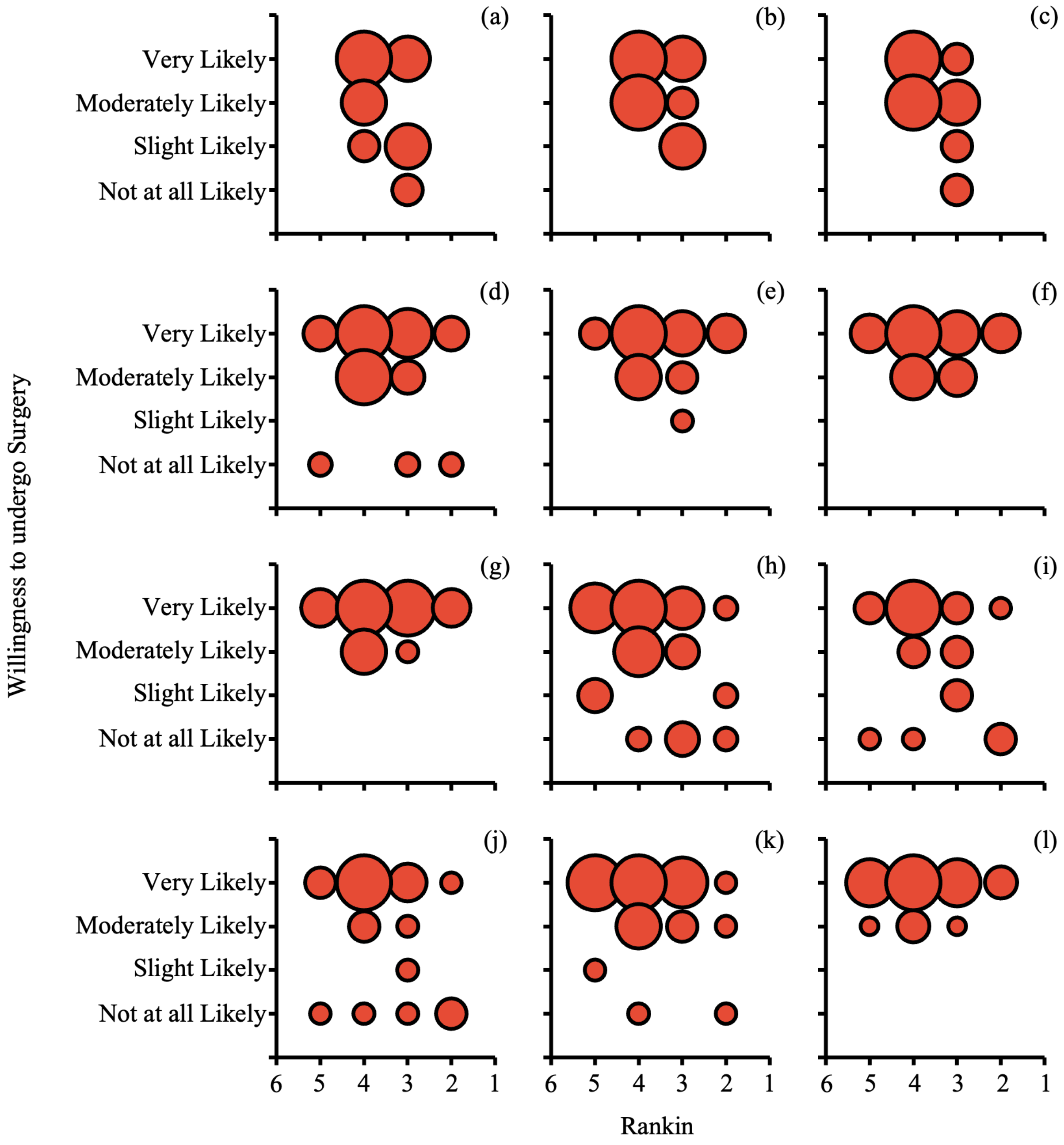

Figure a5: Participant's willingness to undergo surgery to restore different functions given different levels of disability measured by Modified Rankin Scale. Modified Rankin goes from 0 to 6 with 0 being no disability and 6 being dead. "does not apply" was excluded from the bubble charts. Rankin vs. Restoring (a) Grasp and Release (n=28); (b) Grasp and Release + Finger Control (n=28); (c) Grasp and Release + Finger Control + Arm Control (n=30). Rankin vs. Restoring Ability to (d) Stand (n=21); (e) Walk at Constant Speed (n=24); (f) Walk at Various Speeds (n=24); (g) Walk at Various Speeds + Turn (n=26). Rankin vs. Restoring (h) Leg Sensation (n=43); (i) Arm Sensation (n=33); (j) Hand and Finger Sensation (n=35); (k) Bladder Fullness Sensation (n=31); (l) Urination and Defecation Control (n=34).

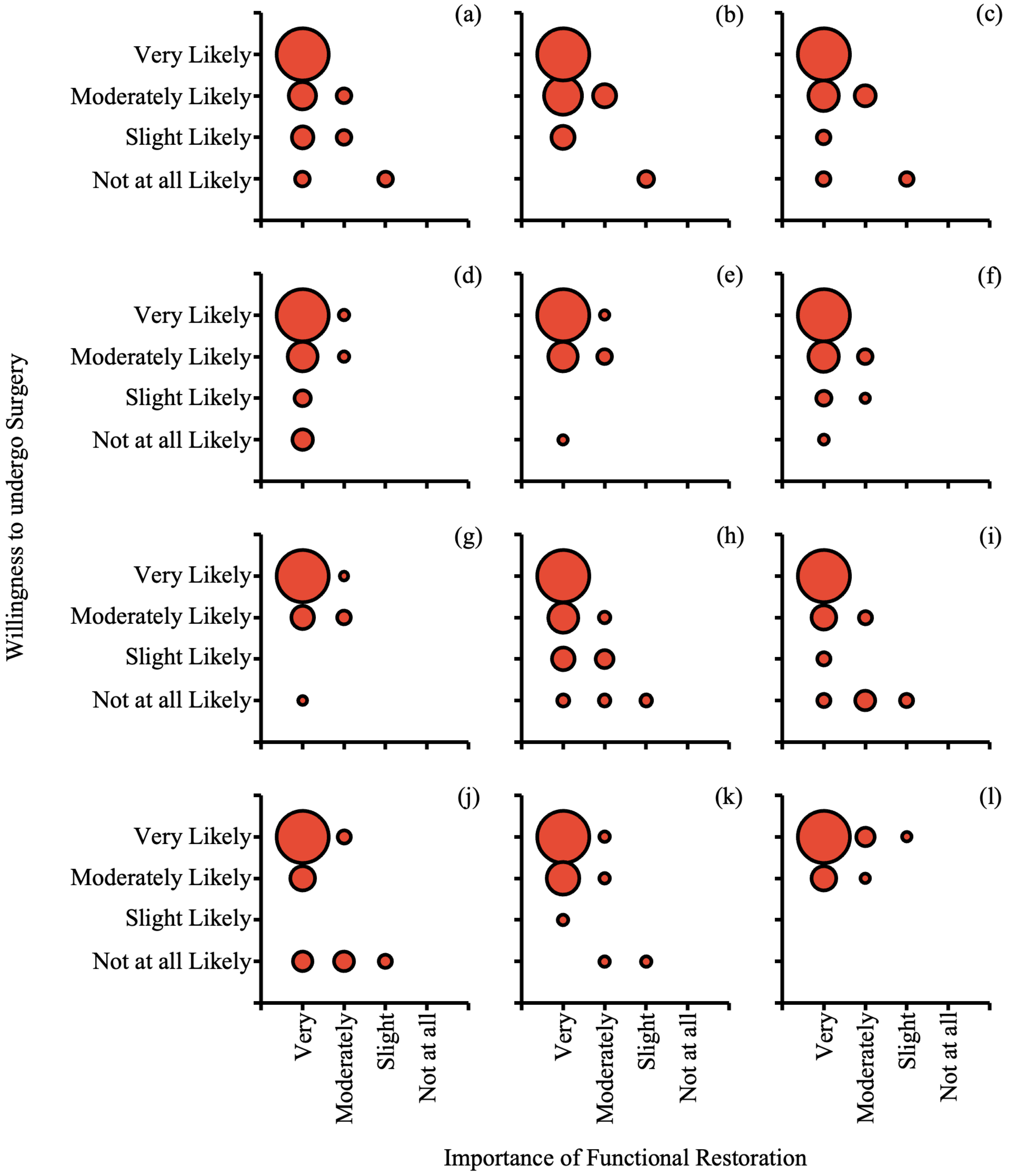

Figure a6: SCI Participant's willingness to undergo surgery to restore different functions given Perceived Importance of Functional Restoration. "does not apply" was excluded from the bubble charts. Importance of Upper Extremity Function vs. Restoring (a) Grasp and Release (n=19); (b) Grasp and Release + Finger Control (n=19); (c) Grasp and Release + Finger Control + Arm Control (n=20). Importance of Lower Extremity Function vs. Restoring Ability to (d) Stand (n=30); (e) Walk at Constant Speed (n=31); (f) Walk at Various Speeds (n=32); (g) Walk at Various Speeds + Turn (n=32). Importance of Sensation vs. Restoring (h) Leg Sensation (n=28); (i) Arm Sensation (n=21); (j) Hand and Finger Sensation (n=21); (k) Bladder Fullness Sensation (n=29); (l) Urination and Defecation Control (n=30).

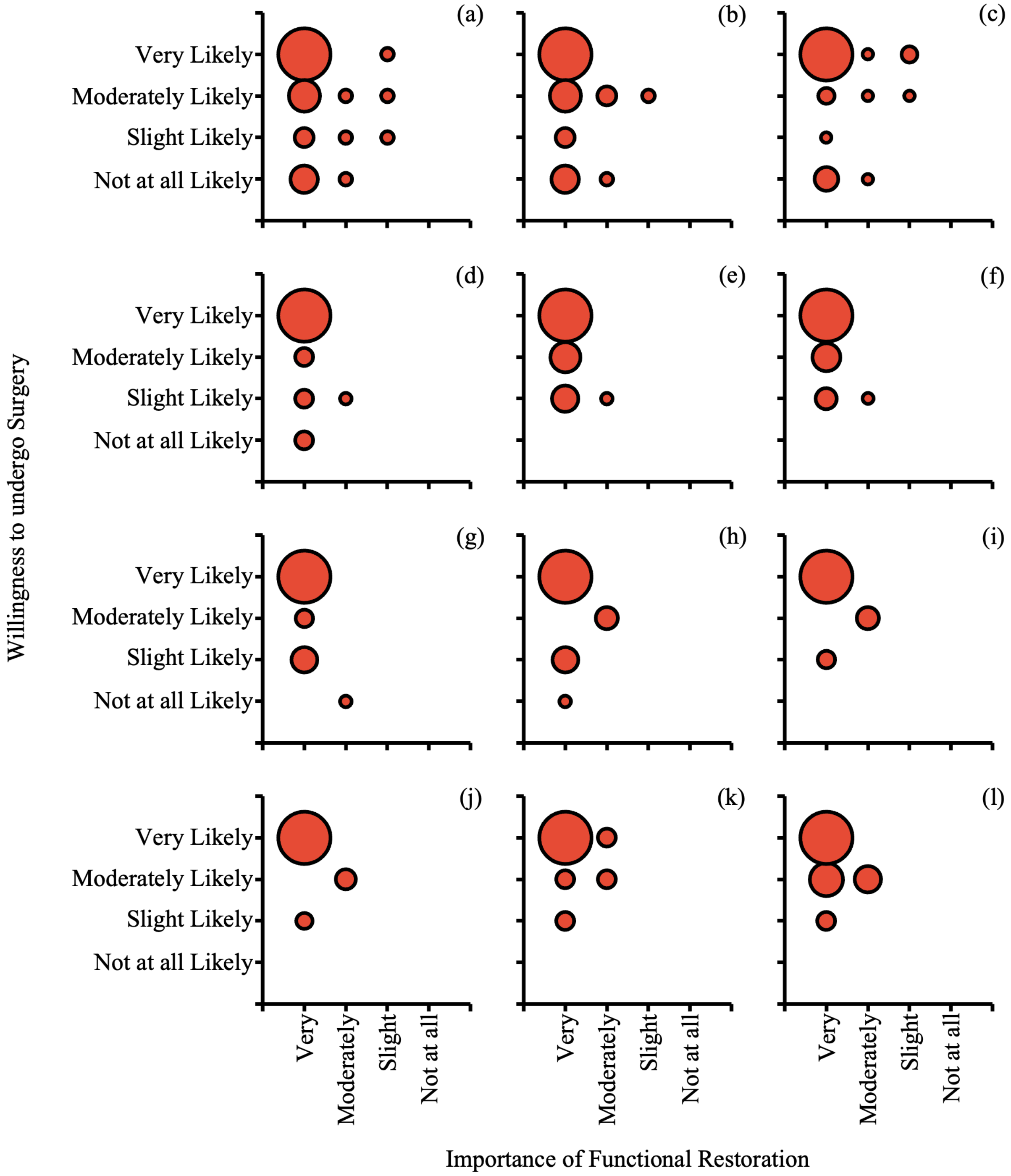

Figure a7: Stroke participant's willingness to undergo surgery to restore different functions given Perceived Importance of Functional Restoration. "does not apply" was excluded from the bubble charts. Importance of Upper Extremity Function vs. Restoring (a) Grasp and Release (n=30); (b) Grasp and Release + Finger Control (n=28); (c) Grasp and Release + Finger Control + Arm Control (n=30). Importance of Lower Extremity Function vs. Restoring Ability to (d) Stand (n=21); (e) Walk at Constant Speed (n=24); (f) Walk at Various Speeds (n=25); (g) Walk at Various Speeds + Turn (n=22). Importance of Sensation vs. (h) Leg Sensation (n=23); (i) Arm Sensation (n=20); (j) Hand and Finger Sensation (n=22); (k) Bladder Fullness Sensation (n=11); (l) Urination and Defecation Control (n=13).

Table S1. Post-hoc demographic analysis of BCI receptiveness using relative risk methodology. RR > 1 indicates the first group (younger, male, high education, high income) was more willing/rated higher importance. *Significant (95% CI excludes 1.0).

Outcome	Comparison	RR	95% CI
Willingness to Undergo Surgery			
Upper Extremity Motor	Age	0.88	[0.66, 1.17]
Upper Extremity Motor	Gender	0.91	[0.70, 1.20]
Upper Extremity Motor	Education	1.03	[0.73, 1.47]
Upper Extremity Motor	Income	1.09	[0.72, 1.64]
Lower Extremity Motor (Walking)	Age	1.01	[0.87, 1.18]
Lower Extremity Motor (Walking)	Gender	1.02	[0.85, 1.24]
Lower Extremity Motor (Walking)	Education	1.17	[0.86, 1.58]
Lower Extremity Motor (Walking)	Income	1.05	[0.95, 1.16]
Leg Sensation	Age	0.74	[0.50, 1.08]
Leg Sensation	Gender	1.14	[0.73, 1.78]
Leg Sensation*	Education	0.76	[0.60, 0.97]
Leg Sensation*	Income	1.46	[1.08, 1.98]
Arm Sensation	Age	0.72	[0.51, 1.01]
Arm Sensation	Gender	1.10	[0.75, 1.61]
Arm Sensation	Education	0.84	[0.69, 1.02]
Arm Sensation*	Income	1.42	[1.04, 1.93]
Hand/Finger Sensation	Age	0.72	[0.51, 1.01]
Hand/Finger Sensation	Gender	1.11	[0.76, 1.62]
Hand/Finger Sensation	Education	0.85	[0.71, 1.02]
Hand/Finger Sensation*	Income	1.36	[1.04, 1.78]
Bladder/Bowel	Age	1.06	[0.95, 1.17]
Bladder/Bowel	Gender	0.96	[0.89, 1.04]
Bladder/Bowel	Education	0.95	[0.86, 1.05]
Bladder/Bowel	Income	1.07	[0.94, 1.23]
Perceived Importance of Functional Restoration			
Upper Extremity Motor	Age	0.97	[0.80, 1.17]
Upper Extremity Motor	Gender	0.94	[0.79, 1.11]
Upper Extremity Motor	Education	0.99	[0.81, 1.21]
Upper Extremity Motor	Income	1.06	[0.84, 1.35]
Lower Extremity Motor	Age	0.96	[0.88, 1.04]
Lower Extremity Motor	Gender	0.97	[0.92, 1.03]
Lower Extremity Motor	Education	1.11	[0.90, 1.37]
Lower Extremity Motor	Income	1.05	[0.96, 1.15]
Sensation	Age	0.94	[0.84, 1.06]
Sensation	Gender	0.97	[0.91, 1.03]
Sensation	Education	1.17	[0.86, 1.58]
Sensation	Income	1.05	[0.95, 1.16]
